# Plasticity manifolds and degeneracy govern circadian oscillations of neuronal intrinsic properties in the suprachiasmatic nucleus

**DOI:** 10.1016/j.isci.2023.106503

**Published:** 2023-03-27

**Authors:** Harshith Nagaraj, Rishikesh Narayanan

**Affiliations:** 1 Cellular Neurophysiology Laboratory, Molecular Biophysics Unit, Indian Institute of Science, Bangalore 560012, India; 2 Undergraduate Program, Indian Institute of Science, Bangalore 560012, India

**Keywords:** Neuroscience

## Abstract

A heterogeneous neuronal population in the suprachiasmatic nucleus (SCN) sustains a cell-autonomous code for circadian time, implemented by firing-rate plasticity involving multiple ion channels. How do SCN neurons undergo stable firing-rate transitions if several ion channels change simultaneously in a heterogeneous neuronal population? Here, we addressed this question by building a heterogeneous population of SCN model neurons, each allowed to undergo one complete circadian cycle through multiple possible routes. We found that SCN neurons could achieve signature electrophysiological characteristics (day-like or night-like) despite pronounced heterogeneity in ion-channel conductances. Furthermore, for any neuron, disparate combinations of ion-channel plasticity yielded valid day-to-night or night-to-day transitions. Finally, nonlinear dimensionality reduction analyses on valid plasticity spaces revealed a low-dimensional plasticity manifold in day-to-night transitions, but not in night-to-day transitions. Our analyses unveil a synthesis of the degeneracy and the plasticity manifold frameworks that provides robustness and flexibility in achieving precise transitions despite widespread heterogeneities.

## Highlights

• Neural heterogeneities critically contribute to robustness of circadian oscillations
• Ion-channel degeneracy: Disparate ionic conductances yield characteristic physiology
• Plasticity degeneracy: Disparate ion-channel changes yield same circadian transitions
• Plasticity manifolds: Simultaneous plasticity in multiple ion channels is structured

## Graphical abstract

**Figure:**
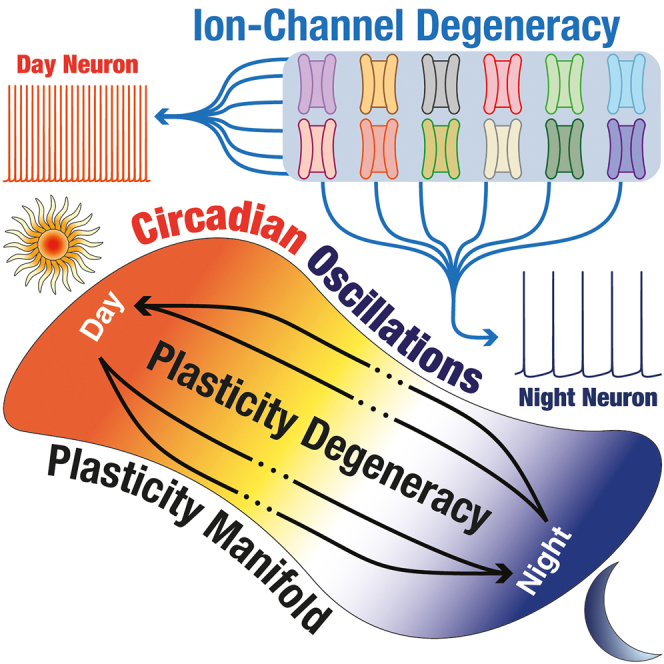

## Introduction

The day-night cycle governs several aspects of the life of organisms on planet Earth. The rotation of the Earth introduces critical changes to several environmental factors including light levels and temperature, which have differential impacts on different organisms. As these environmental changes set constraints on foraging and other behaviors related to survival, organisms have evolved several adaptation strategies to align their behavior and physiology to the day-night cycle. The suprachiasmatic nucleus (SCN) is the site of the master circadian clock of the mammalian brain that plays a central role in this adaptation, sustaining a neural code for circadian time through the firing rate of constituent neurons. Periodic changes in intrinsic electrophysiological properties (including firing rate) are cell-autonomous, maintained by a transcription-translation feedback loop (TTFL). The TTFL is tightly intercoupled with neural activity and several molecular signaling cascades, with the rhythm entrained to the external day-light cycle by retinal projections to the SCN.^1^^,^^2^^,^^3^^,^^4^^,^^5^^,^^6^^,^^7^^,^^8^

Cell-autonomous oscillations in neuronal intrinsic electrophysiological properties are mediated by changes in a subset of ion channels expressed in SCN neurons.^1^^,^^9^^,^^10^^,^^11^^,^^12^^,^^13^^,^^14^^,^^15^^,^^16^^,^^17^^,^^18^^,^^19^^,^^20^ These circadian oscillations are precisely sustained despite recruiting recurring changes in multiple ion channels and despite pronounced neuron-to-neuron variability in the intrinsic electrophysiological properties of SCN neurons. The question of how plasticity in these ion channels governs neuronal intrinsic electrophysiological properties through circadian oscillations has remained unexplored, especially considering the high degree of neuron-to-neuron variability observed in SCN neurons. Specifically, how are SCN neurons able to undergo precise circadian transitions in their intrinsic electrophysiological properties despite pronounced variability in the underlying biophysical parameters? Why do so many different ion channels undergo changes during circadian oscillations, when it could have been simpler to design a regulatory mechanism that allows plasticity in a single ion-channel subtype? How does the system undergo stable transitions and maintain precision in intrinsic electrophysiological properties spanning the day-night cycle if several ion channels change concomitantly in a heterogeneous neuronal population?^1^^,^^7^^,^^21^

**Figure 1:**
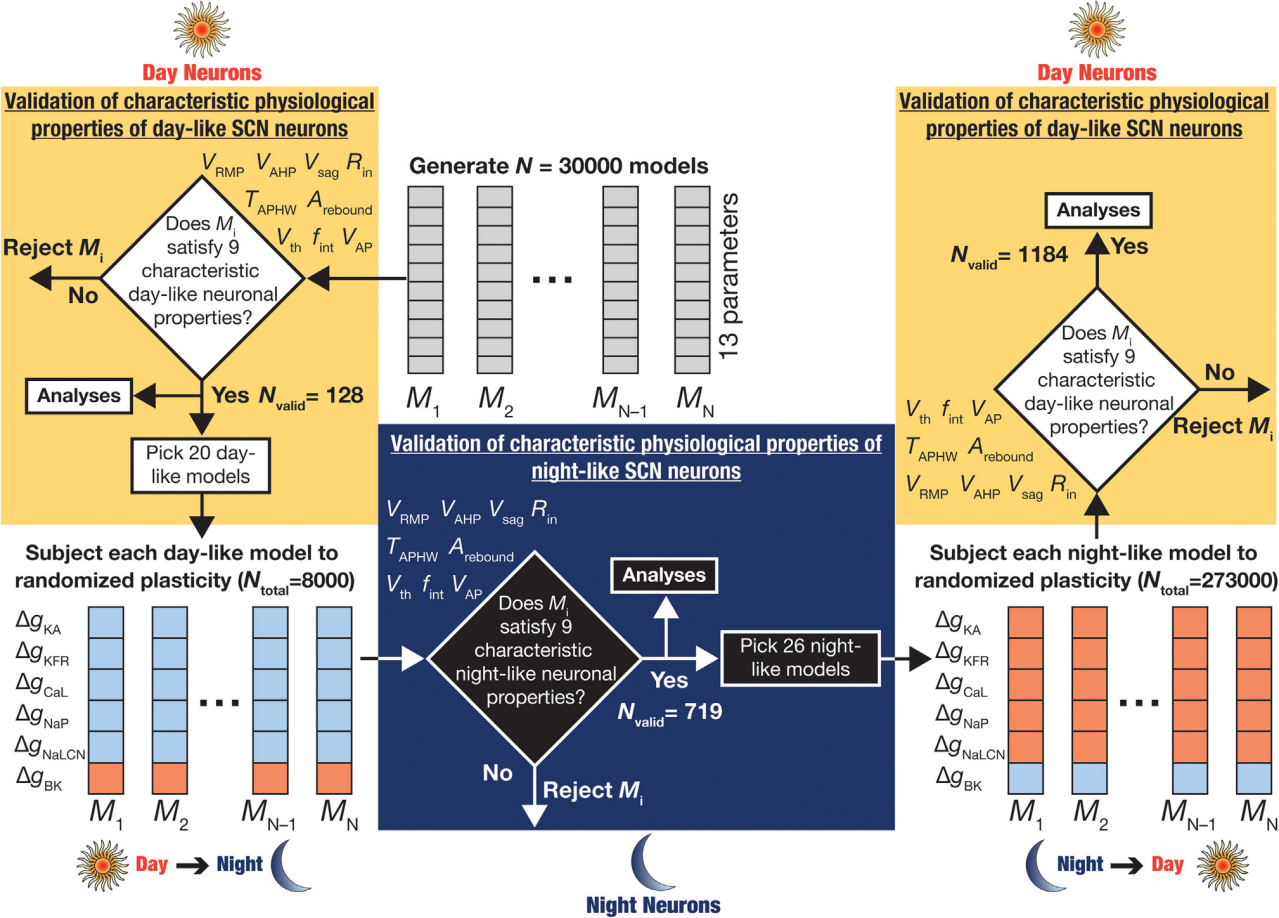
Flowchart illustrating the overall methodological plan for assessing ion-channel degeneracy and plasticity manifolds in circadian oscillations in SCN neurons *Left,* The first set of day-like neurons were generated by a *de novo* unbiased search involving 13 different parameters involving 30000 neuron models. Of these, 128 were found to show valid day-like physiological properties. These models were assessed for the expression of ion-channel degeneracy and heterogeneities. As a second step, 20 of these 128 day-like models were picked and subjected to day-to-night transitions that involved plasticity in six different ion channels in electrophysiologically determined directions (red implies increase, blue implies reduction). *Center,* Models subjected to randomized plasticity ($N_{total}$ = 8000 total random transitions) were validated with night-like measurements from SCN neurons, and 719 models derived from the 20 day-like models were found to be valid. These models were assessed for manifestation of ion-channel degeneracy and heterogeneities. The specific combinations of ion-channel plasticity (from respective day-like neurons) that resulted in valid night-like models were subjected to dimensionality reduction analysis to determine the presence of structured plasticity manifolds in the day-to-night transitions. *Right,* As a third step, 26 of these 719 night-like models were picked and subjected to night-to-day transitions that involved plasticity in six different ion channels in electrophysiologically determined directions (red implies increase, blue implies reduction). Models subjected to randomized plasticity ($N_{total}$ = 273,000 total random transitions) were validated with day-like measurements from SCN neurons, and 1184 models derived from the 26 night-like models were found to be valid. These models were assessed for manifestation of ion-channel degeneracy and heterogeneities. The specific combinations of ion-channel plasticity (from respective night-like neurons, as well as the original day-like neurons from step 1) that resulted in valid day-like models were subjected to dimensionality reduction analysis to determine the presence of structured plasticity manifolds in these transitions.

In this computational study, we address these important questions by first generating a population of mathematical models of day-like SCN neurons (Figure 1). These mathematical models were constrained to match biophysical properties of SCN neurons and were validated against several characteristic electrophysiological measurements. We then subjected this day-like model population to an entire cycle of circadian oscillations, not by hand tuning ion-channel plasticity, but by searching for *multiple* valid transitions through an unbiased search of the plasticity space in each model. These unbiased searches were performed independently for the day-to-night and subsequent night-to-day transitions for multiple day-like and night-like models, respectively. We constrained the permitted plasticity space for day-to-night and night-to-day transitions both in terms of the subset of channels that were allowed to change and the direction in which they could change.^1^^,^^9^^,^^10^^,^^11^^,^^12^^,^^13^^,^^14^^,^^15^^,^^16^^,^^17^^,^^18^^,^^19^^,^^20^^,^^22^ Together, our approach involved independent and unbiased searches, first involving the parametric space for a valid *population* of day-like models, and subsequently on the plasticity space for *populations* of valid day-to-night and night-to-day transitions (Figure 1).

Our populations-based approach allowed for exploring the impact of heterogeneities in both the parametric and plasticity spaces on the circadian oscillations of SCN intrinsic electrophysiological properties. Our analyses provided answers to the questions posed above within the elegant frameworks of degeneracy and plasticity manifolds. Degeneracy is defined as the ability of disparate structural components to elicit similar functional outcomes.^23^^,^^24^^,^^25^^,^^26^^,^^27^ Plasticity manifolds refer to the presence of strong constraints on the ability of different components to change together.^28^ The synthesis of two frameworks provided important insights about circadian oscillations in SCN neurons. First, we show that SCN neurons could achieve signature electrophysiological characteristics despite pronounced heterogeneity in ion-channel conductances and calcium kinetics. It was not essential to maintain biophysical parameters at a precise value for achieving characteristic electrophysiological signatures of SCN neurons under day or night conditions. This ion-channel degeneracy was observed in the valid day-like population of SCN models and in the models obtained after valid day-to-night and night-to-day transitions as well.

Second, our approach to look for *multiple* valid transitions from any given SCN model showed that there are several routes to achieve valid day-to-night or night-to-day transitions from any day-like or night-like neuron, respectively. Specifically, it was not essential that specific conductances had to change by precise values for obtaining valid transitions. As disparate combinations of ion-channel plasticity could yield the same functional transition, we refer to this as *plasticity degeneracy* in SCN neurons. The ability to change multiple ion channels concomitantly allows flexibility in each neuron to take one of several routes to achieve valid transitions, rather than being constrained by changes to one specific ion channel. These analyses also showed that given the inherent heterogeneities in individual neurons, the plasticity routes to achieve valid transitions were dependent on the specific neuron under consideration. Together, the ability of multiple ion channels to change during circadian oscillations provides robustness and flexibility to effectively achieve precise transitions despite widespread heterogeneities in ion-channel expression through plasticity degeneracy.

Finally, we posed the question of how SCN neurons maintained stability through continual transitions if so many ion channels were allowed to change simultaneously. An elegant solution to this problem is to constrain the multi-dimensional plasticity space involving several ion channels to a structured low-dimensional manifold so that individual ion channels do not undergo arbitrary plasticity resulting in instability.^28^ We performed nonlinear dimensionality reduction analyses on the valid day-to-night and night-to-day populations of transitions to explore if valid transitions were constricted within *plasticity manifolds*. Strikingly, we found the manifestation of a low-dimensional manifold in the day-to-night transitions, but not in the night-to-day transitions. These observations demonstrated that the concomitant changes in multiple ion channels are not arbitrary but follow a structure that provides a substrate for stability in achieving valid circadian oscillations.

## Results

The overall goal of this study was to explore heterogeneities in and constraints on ion channels and their plasticity in the emergence of SCN neurons endowed with characteristic physiological properties and signature circadian oscillations in their intrinsic physiology (Figure 1). As the use of single hand-tuned models does not capture the heterogeneity that is characteristic of all biological systems, we generated a population of neurons that was endowed with characteristic biophysical properties (Figure S1) and satisfied signature electrophysiological properties of SCN neurons (Figure S2). We used this population of neurons to assess heterogeneity of measurements, degeneracy in parametric space, and the evolution of the parametric and measurement space as the population was subjected to day-to-night followed by night-to-day transitions (Figure 1).

### Ion-channel degeneracy in SCN neurons manifesting day-like characteristics

We built a single-compartmental, conductance-based mathematical model of an SCN neuron, incorporating 12 active and passive conductances with characteristics derived from biophysical measurements from SCN neurons (Figure S1). An unbiased stochastic search algorithm was employed spanning 13 parametric values, which accounted for these channel conductances as well as cytosolic calcium decay (Table 1). A randomized population of 30,000 unique model neurons was generated by sampling this 13-dimensional parametric space and a set of 9 electrophysiological measurements were recorded from each model (Table 2; Figure S2). These measurements were validated by comparing model measurements against established electrophysiological bounds characteristic of day-like SCN neurons (Table 2). A small subset of 128 models (128/30000 = 0.4%) satisfied all 9 day-like measurement constraints (Table 2) and were declared as valid day-like SCN neurons.

**Table 1:** Range of parameters used in generating the model populations

	Parameter, symbol (Unit)	Lower bound	Upper bound
1	Maximal conductance of KFR channels, ${\bar{g}}_{KFR}$ (mS/cm^2^)	0.1	1
2	Maximal conductance of KSR channels, ${\bar{g}}_{KSR}$ (mS/cm^2^)	0.1	1
3	Maximal conductance of KA channels, ${\bar{g}}_{KA}$ (mS/cm^2^)	0.01	0.1
4	Maximal conductance of NaF channels, ${\bar{g}}_{NaF}$ (S/cm^2^)	0.05	0.5
5	Maximal conductance of NaP channels, ${\bar{g}}_{NaP}$ (mS/cm^2^)	0.01	0.1
6	Maximal conductance of CaL channels, ${\bar{g}}_{CaL}$ (mS/cm^2^)	0.1	1
7	Maximal conductance of CaP channels, ${\bar{g}}_{CaP}$ (mS/cm^2^)	0.1	1
8	Maximal conductance of SK channels, ${\bar{g}}_{SK}$ (μS/cm^2^)	1	10
9	Maximal conductance of BK channels, ${\bar{g}}_{BK}$ (S/cm^2^)	0.01	0.1
10	Maximal conductance of HCN channels, ${\bar{g}}_{HCN}$ (μS/cm^2^)	5	50
11	Maximal conductance of NaLCN channels, ${\bar{g}}_{NaLCN}$ (μS/cm^2^)	5	50
12	Specific membrane resistance, $R_{m}$ (kΩ.cm^2^)	20	40
13	Calcium decay time constant, $\tau_{Ca}$ (s)	1.75	2.24

**Table 2:** Bounds on intrinsic electrophysiological measurements used for validating the day-like and night-like neurons

	Measurement (Unit)	Day neurons		Night neurons	
		Lower bound	Upper bound	Lower bound	Upper bound
1	$V_{RMP}$ (mV)	−60	−52	−75	−65
2	$R_{in}$ (GΩ)	0.768	1.812	0.5	1.5
3	$V_{AP}$ (mV)	70	–	70	–
4	$V_{th}$ (mV)	−44.7	−36.5	–	–
5	$T_{APHW}$ (ms)	1	2	1	2
6	$V_{AHP}$ (mV)	−25.8	−16.8	−31	−17
7	$A_{rebound}$ (mV.ms)	−510	966	−510	966
8	$f_{\mathrm{int}}$ (Hz)	3	7	0	2
9	$V_{sag}$ (mV)	2	10	2	16

**Figure 2:**
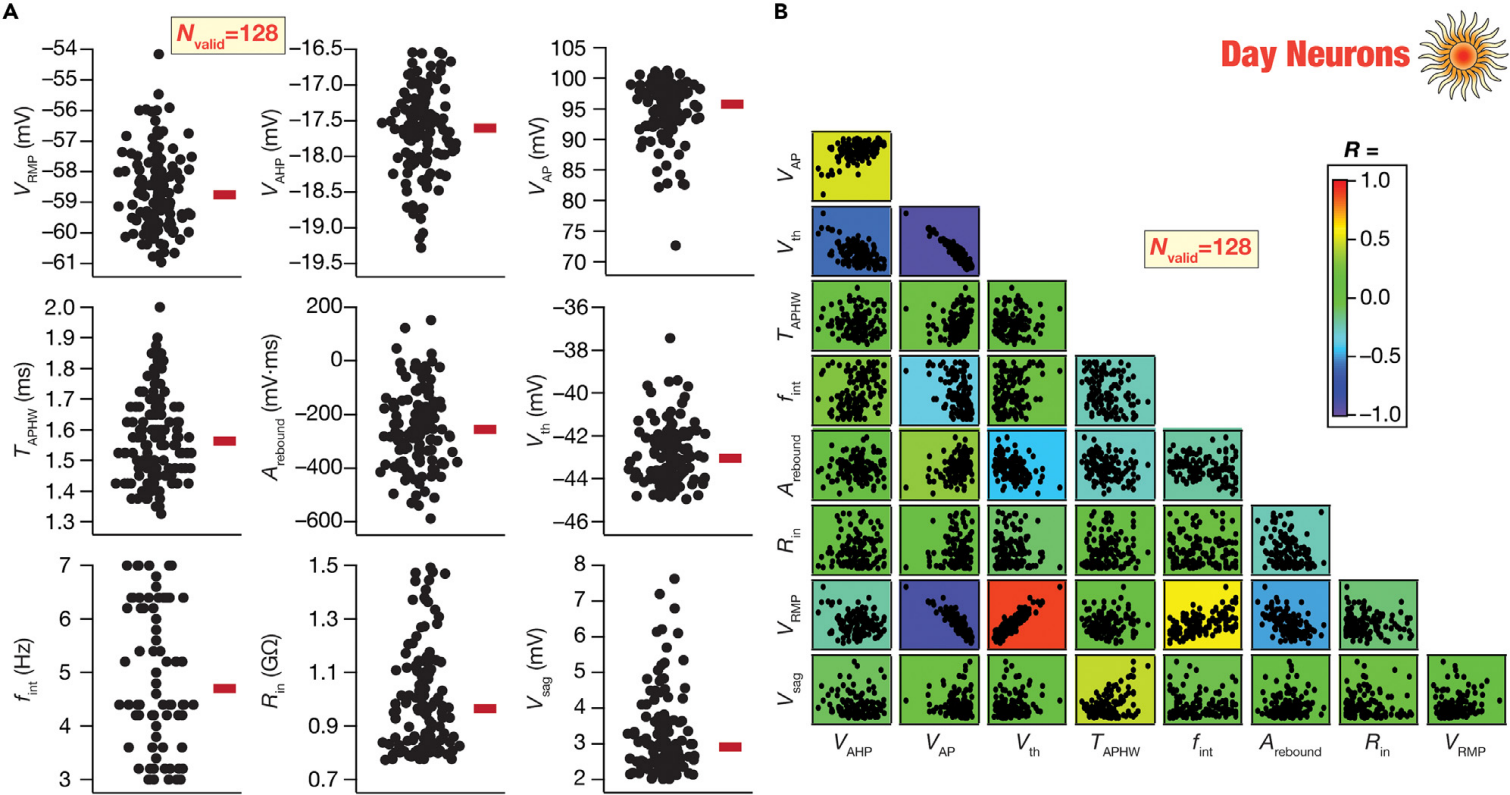
Heterogeneous distribution of measurements from day-like SCN neurons (A) Bee-swarm plots of measurements from 128 different SCN neurons exhibiting day-like phenotype. Red bars represent the median values. The neurons were obtained using the MPMOSS algorithm and were validated using the respective electrophysiological bounds (Table 1). The 128 models shown here were obtained from 30,000 runs of the MPMOSS algorithm and exhibited pronounced heterogeneities within their respective bounds (Table 1). (B) Pairwise correlations between the different measurements used to characterize and validate day-like neuronal models. The scatterplots are overlaid on top of a color-coded matrix showing the respective Pearson’s correlation coefficient values.

The search and validation process yielded a heterogeneous population of valid day-like SCN models that reflected electrophysiological heterogeneities of SCN neurons (Figure 2A; Table 2). We asked if the different measurements associated with the 128 valid models manifested pairwise correlations. We found that most of these pairwise correlations were weak (Figure 2B), implying that these 9 measurements were characterizing distinct aspects of SCN physiology. The few strong pairwise correlations observed were expected because of their cross-dependencies arising either from the inherent nature of measurement or because of the common set of ion channels that are expected to govern these properties in individual neurons. For instance, action potential threshold, resting membrane potential, AHP potential, and action potential amplitude showed strong correlations because of how these measurements are performed in a spontaneously firing neuron (Figure S2). The firing rate $f_{\mathrm{int}}$ was expectedly higher in neurons with depolarized resting membrane potential, manifesting as a strong positive correlation (Figure 2B).

**Figure 3:**
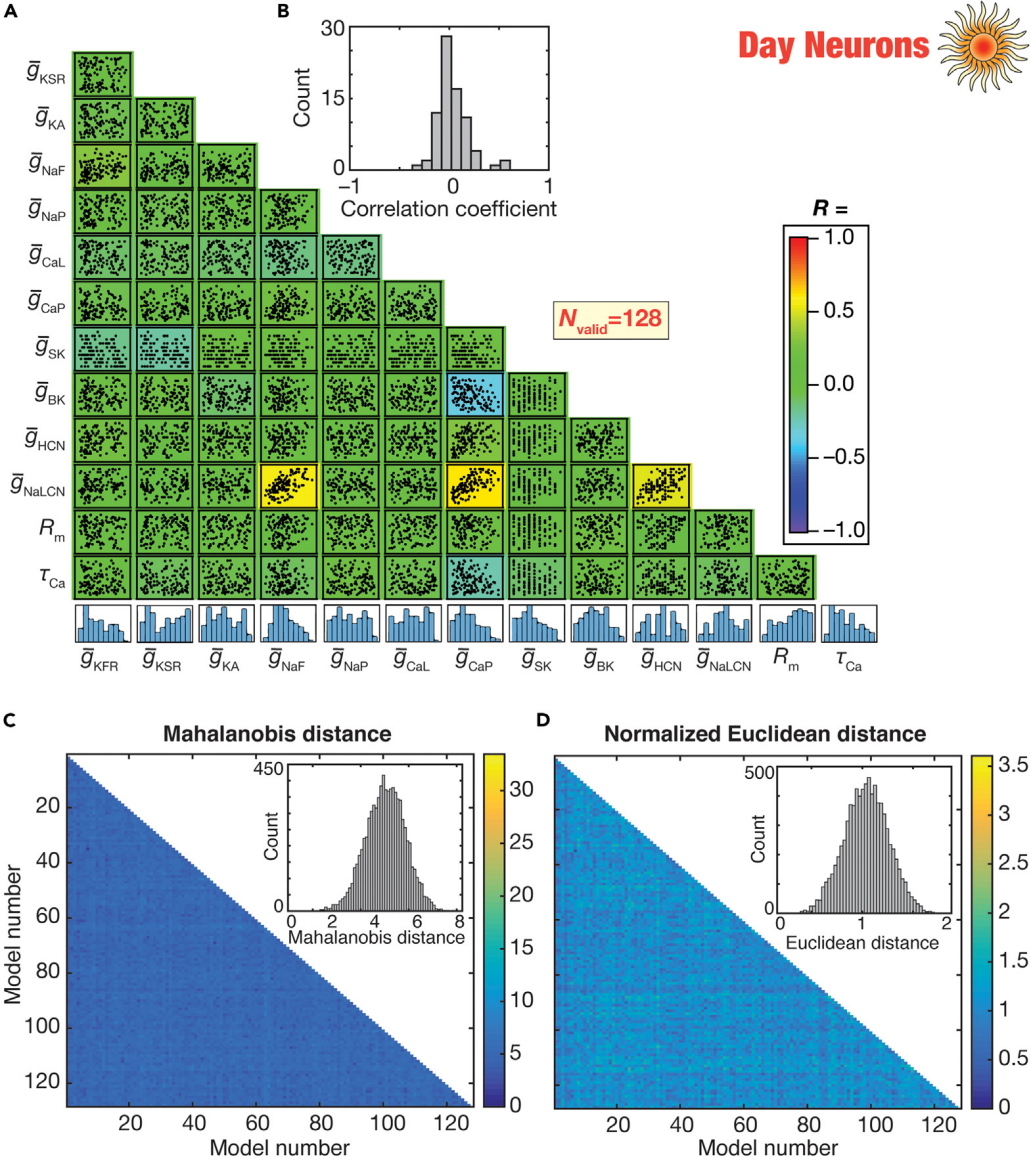
The day-like neuronal model population manifested parametric heterogeneity and weak pairwise correlations in the parametric space (A) Scatterplot matrix between the different parametric values of the 128 day-like SCN models that are shown in Figure 2. The background color represents the value of the Pearson correlation coefficient between the different parameters. Histograms representing the distributions of individual parameters are shown on the bottom-most row. (B) Histogram representing the distribution of the correlation values shown in panel (A). Note that all absolute values of the correlation coefficient (*R*) were <0.6, implying an *R*^2^ value less than 0.36, indicating weak pairwise correlations between parametric distributions. (C and D) Heterogeneities in model parameters quantified with Mahalanobis (C) or normalized Euclidean (D) distances. The matrices represent the pairwise distance between the parametric vectors defining the 128 models. Insets show the histogram of the values in the distance matrix.

How constrained were the parametric distributions in these models that exhibited characteristic signatures of day-like SCN neurons? Were they clustered around a specific location or were they distributed across a large swath of the allowed range (Table 1)? To address these, we first picked 7 models that showed similar physiological measurements (Figure S3A–F). We found that the parametric distributions of these models showing similar functions spanned their entire ranges (Figure S3G), thus providing a line of evidence on the manifestation of ion-channel degeneracy in these models. Specifically, these illustrative examples provide evidence that it is not essential to maintain the conductances of different channels at specific levels for achieving the characteristic physiological properties of day-like SCN neurons. We assessed the distributions of parameters across all 128 valid models and found them to show widespread distributions spanning the entire stretch of their respective parametric ranges (Figure 3A, histograms; Table 1).

How did models achieve characteristic physiology despite such widespread distribution of underlying parameters? Was variability in the expression of one ion-channel subtype compensated by specific differences in another conductance value? To address this, we computed pairwise correlations between the different parameters across the 128 valid models (Figures 3A and 3B). We found most pairwise correlations to be weak (Figures 3A and 3B), suggesting that variability in one channel parameter was not compensated by variability in another, but because of synergistic interactions between several ion channels defining these model populations. Specifically, strong pairwise correlations between ion-channel pairs would have pointed to the presence of functionally equivalent pairs of ion channels compensating for each other in a correlated fashion. The weak pairwise correlations illustrate the absence of such pairwise local compensations, instead pointing to synergy across all ion channels in achieving ion-channel degeneracy. Importantly, we also found that pairwise distances between these models in the 13-dimensional space, computed either through Mahalanobis distance (Figure 3C) or normalized Euclidean (Figure 3D) distance, showed large distances between these models, thus ruling out clustering of these models in the parametric space.

The results of linear dimensionality reduction analysis on the measurement and parametric spaces of these 128 neurons, using principal component analysis (PCA), did not show any clustering (Figure S4). The widespread distribution of model parameters or weak pairwise correlations or large pairwise distances does not imply that any arbitrary combination of these 13 parameters can yield valid day-like SCN model neurons. A vast majority (29872/30000 = 99.57%) of the parametric combinations spanning this exact same range were in fact rejected during the validation process yielding only 128 valid models out of the 30,000 random samples tested. Thus, these observations imply that there are disparate, yet *specific combinations* of parameters, which do not show discernable structure in pairwise relationships or in individual distributions, that can yield similar characteristic physiological properties observed in SCN neurons. Together, our analyses involving a heterogeneous population of day-like SCN neurons manifesting widespread parametric variability provide evidence for the manifestation of ion-channel degeneracy in the concurrent emergence of several characteristic properties of SCN neurons. There was no clustering of or pairwise correlations across parametric values associated with the model population, thus emphasizing a global (rather than pairwise local) structure in the parametric subspace that yielded valid models.

### Plasticity degeneracy in achieving valid day-to-night transitions unveiled ion-channel degeneracy in night-like neurons

**Figure 4:**
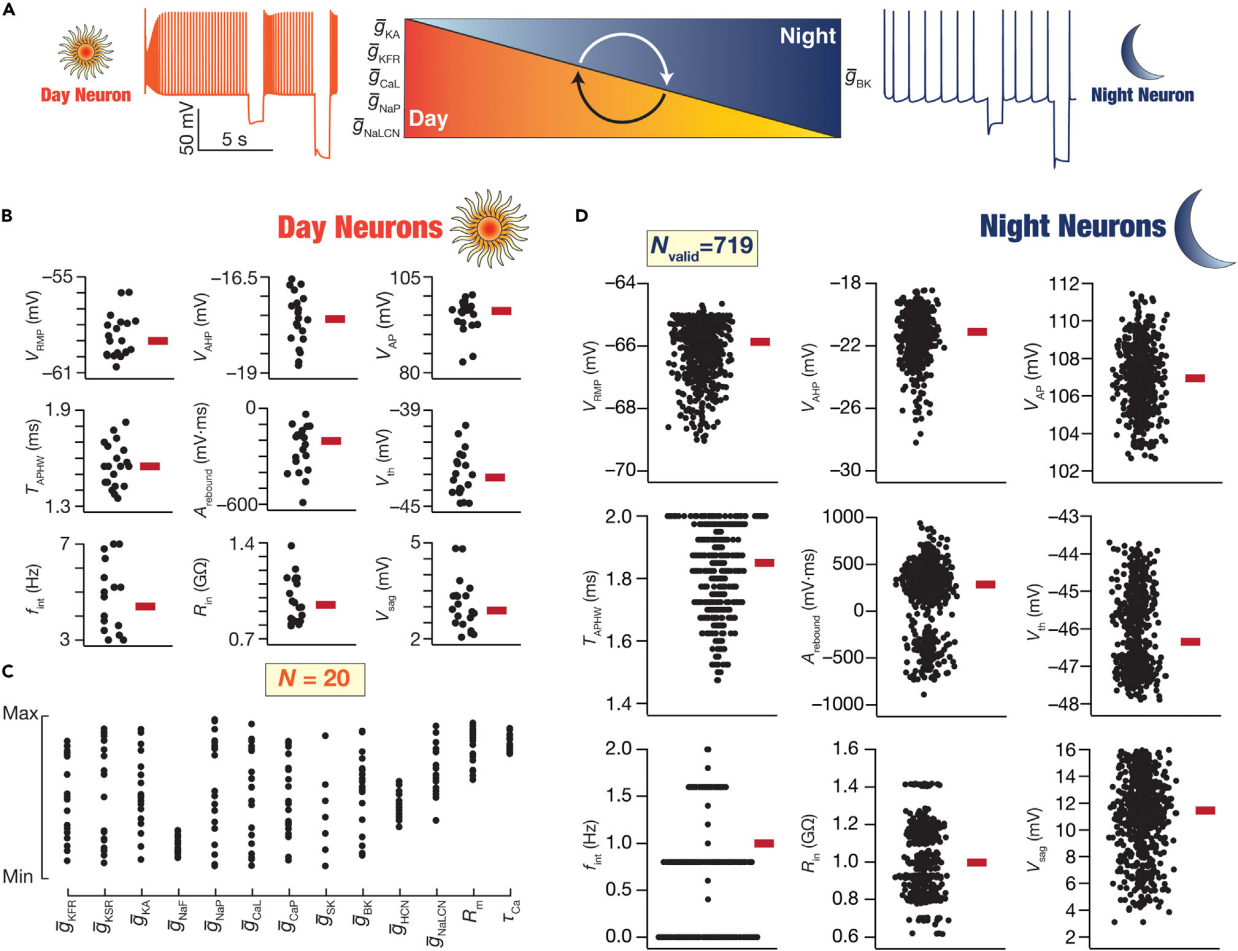
Heterogeneous distribution of measurements from night-like SCN neurons derived through unbiased search of transitions from day-like neurons (A) Schematic diagram of day-to-night transitions, along with representative voltage traces from day-like and night-like models of SCN neurons. Electrophysiologically observed circadian transitions in 6 ion-channel conductances are shown. 5 conductances ($g_{NaLCN}$, $g_{NaP}$, $g_{CaL}$, $g_{KFR}$, and $g_{KA}$) show high values during daytime, whereas $g_{BK}$ manifests low values during daytime. (B and C) Bee-swarm plots of measurements (B) and parameters (C) of the 20 day-like SCN neuron models that were subjected to the day-to-night transition. The transition was implemented through a modified MPMOSS algorithm that was employed to perform an unbiased search on the plasticity space. The plasticity space accounted for the physiological direction of changes in the six ion channels (shown in panel (A)) that are known to undergo plasticity during circadian oscillations. The widespread distribution of the measurements and the parameters of the 20 day-like neurons may be noted. (D) Bee-swarm plots of the measurements from different SCN night-like neurons derived from the 20 day-like neurons. Red bars represent the median values. The validation process for obtaining night-like neurons employed established electrophysiological bounds on each measurement (Table 2). The number of valid neurons was 719 for subthreshold measurements and firing rate but was 578 for action potential measurements corresponding to the 578 intrinsically active neurons. Action potential measurements were not feasible from intrinsically silent neurons given our measurement protocols.

A well-studied signature characteristic of SCN neurons is their ability to undergo circadian modulation in their intrinsic properties, manifesting different electrophysiological characteristics during day vs. night periods. These changes in electrophysiological properties are mediated by changes in a specific subset of ion channels, with channel-specific directions in such changes observed during night-to-day and day-to-night transitions. Specifically, there are lines of evidence for reductions in KA, KFR, CaL, NaP, and NaLCN channel conductances and a concurrent increase in BK conductance during day-to-night transitions.^11^^,^^12^^,^^13^^,^^14^^,^^15^^,^^16^^,^^17^ The direction of changes of this subset of channels is opposite for the night-to-day transitions, with the cycle repeated over the circadian period (Figure 4A).

Although the direction of changes in each channel subtype is known, the question of whether such transitions require *unique* magnitude of changes in these individual channels is unanswered. In addition, given the observed heterogeneities in SCN neurons recorded during day periods, how do different day neurons endowed with disparate ion-channel composition achieve transitions toward achieving night-like characteristics? To address these questions, we picked 20 day-like models that manifested heterogeneities in the measurements (Figure 4B) and underlying channel conductances (Figure 4C). We performed a stochastic search on the *plasticity space* involving changes in the subset of ion channels mentioned above for day-to-night (Figure 1; Table 3) transitions, confined to the experimentally determined direction of change for each ion-channel subtype (Table 3; Figure 4A).

**Table 3:** Range of plasticity parameters used in the day-to-night transitions

	Parameter (Unit)	Range of Δg	Relation between $g_{day}$ and $g_{night}$
1	${\bar{g}}_{KFR}$ (mS/cm^2^)	(0,1)	$g_{Night}^{KFR}=g_{Day}^{KFR}\;\left(1-\Delta g\right)$
2	${\bar{g}}_{KA}$ (mS/cm^2^)	(0,1)	$g_{Night}^{KA}=g_{Day}^{KA}\;\left(1-\Delta g\right)$
3	${\bar{g}}_{NaP}$ (mS/cm^2^)	(0,1)	$g_{Night}^{NaP}=g_{Day}^{NaP}\;\left(1-\Delta g\right)$
4	${\bar{g}}_{CaL}$ (mS/cm^2^)	(0,1)	$g_{Night}^{CaL}=g_{Day}^{CaL}\;\left(1-\Delta g\right)$
5	${\bar{g}}_{BK}$ (S/cm^2^)	(0,10)	$g_{Night}^{BK}=g_{Day}^{BK}\;\left(1+\Delta g\right)$
6	${\bar{g}}_{NaLCN}$ (μS/cm^2^)	(0,1)	$g_{Night}^{NaLCN}=g_{Day}^{NaLCN}\left(1-\Delta g\right)$

For each of the 20 day-like neurons, we randomly sampled the 6-dimensional plasticity space involving sign-enforced changes in ion channel densities (Table 3). We subjected individual models to this randomized plasticity by altering the respective channel densities as per the generated sample. We measured the 9 electrophysiological properties of the models after they underwent plasticity and validated them against night-like characteristics of SCN neurons (Table 2). Plasticity parameters that yielded valid night-like neurons were declared as valid day-to-night transitions. For each of the 20 day-like neurons, we generated 200 to 1000 random samples on the plasticity space (Table 3) with a goal of generating at least 20 (max 67 from a day-like neuron) valid day-to-night transitions from each day-like neuron. This process together yielded 719 valid day-to-night transitions from a total of 8000 random transitions that were generated, with all neurons that were declared valid showing night-like characteristics (Figure 4D; *cf*. Table 2). Importantly, these 719 night-like neurons manifested pronounced heterogeneities within the valid measurement ranges (Figure 4D), thus providing us with a heterogeneous population of night-like neurons for further analyses.

From the measurements perspective, as the intrinsic firing rate is lower in night-like neurons compared to their day-like counterparts (Table 2), we found that 141 of these neurons were not spontaneously active with the remaining 578 neurons showing low spontaneous firing (Figure 4D; *cf*. Figure 2A for day-like neurons). To maintain consistency, we measured action potential properties from spontaneous action potential firing and therefore do not report action potential properties for the silent neurons. We computed pairwise correlations between measurements across the intrinsically active (Figure S5A) and silent (Figure S5B) neurons separately and found mostly weak correlations between these measurements. As mentioned earlier, the few measurements that showed strong correlations were largely owing to the relationships between the way these measurements were obtained.

**Figure 5:**
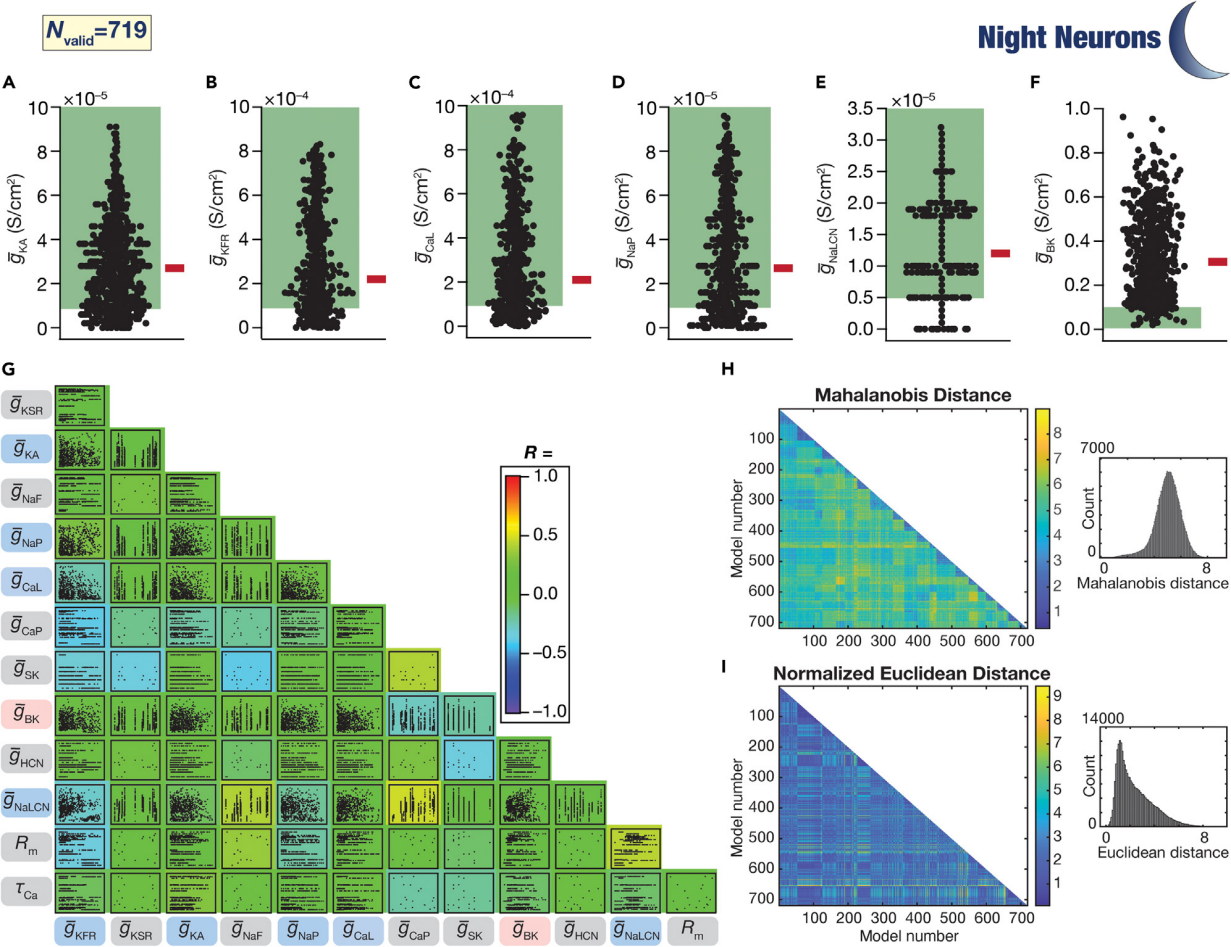
Heterogeneous distribution and weak pairwise correlations of parameters from night-like SCN neurons derived from day-like neurons (A*–*F) Bee-swarm plots of the parameters from 719 different SCN night-like neurons shown in Figure 4. Red bars represent the median values. Green shaded portions represent the set range of the respective parameters in the original day-neuron population (Table 1). (G) Scatterplot matrix between the different parametric values of the 719 night-like SCN models shown in Figure 4. The background color represents the value of the Pearson correlation coefficient between the different parameters. Ion channels represented with blue and red backgrounds show reductions and increases during day-to-night transitions, respectively. Parameters with gray background do not undergo any change during day-to-night transitions, and thus do not change from their respective day neurons. (H and I) Heterogeneities in model parameters quantified with Mahalanobis (H) or normalized Euclidean (I) distances. The matrices represent the pairwise distance between the parametric vectors defining the 719 models. Insets show the histogram of the values in the distance matrix.

The parameters associated with the ion-channel subtypes that were allowed to change during day-to-night transitions manifested widespread heterogeneities, even expanding the ranges allocated (Table 1) for the original day-like model neurons (Figure 5A–F). Notably, although these 719 night-like neurons were derived from merely 20 day-like neurons by allowing only 6 of the 13 original parameters to change during the transition, there were no strong pairwise correlations between the different parameters that governed these model neurons (Figure 5G). Finally, these models also showed large distances among themselves with both Mahalanobis (Figure 5H) and normalized Euclidean (Figure 5I) distance metrics. The results of linear dimensionality reduction analysis on the parametric spaces of these 719 neurons using PCA are shown in Figure S6. The widespread parametric distributions or the lack of pairwise relationships between model parameters should not be misconstrued that any arbitrary value of these conductances or any magnitude of sign-enforced plasticity associated with these 6 channels would yield valid day-to-night transitions or valid night models. It should be noted that most transitions (7281 of 8000 = 91%) were rejected in the stochastic search process that spanned this very plasticity space, and only specific *combinations* of plasticity yielded valid night models. Thus, the emphasis should be on the global structure in the plasticity space whereby specific combinations of disparate plasticity profiles yielded neurons with similar functional profiles.

Together, these analyses unveiled two important conclusions: (i) **ion-channel degeneracy**: disparate ion-channel combinations, with no strong pairwise relationships in channel expression profiles, can yield similar characteristic night-like neurons; and (ii) **plasticity degeneracy**: combinations of very different magnitudes of sign-enforced changes involving 6 disparate ion channels can yield valid and functionally equivalent day-to-night transitions. Our analyses show that the manifestation of ion-channel and plasticity degeneracy ensure that day-to-night transitions did not require unique magnitude of changes in individual channels undergoing plasticity for achieving valid night neurons.

### Ion-channel plasticity associated with day-to-night transitions were constrained by a low-dimensional plasticity manifold

**Figure 6:**
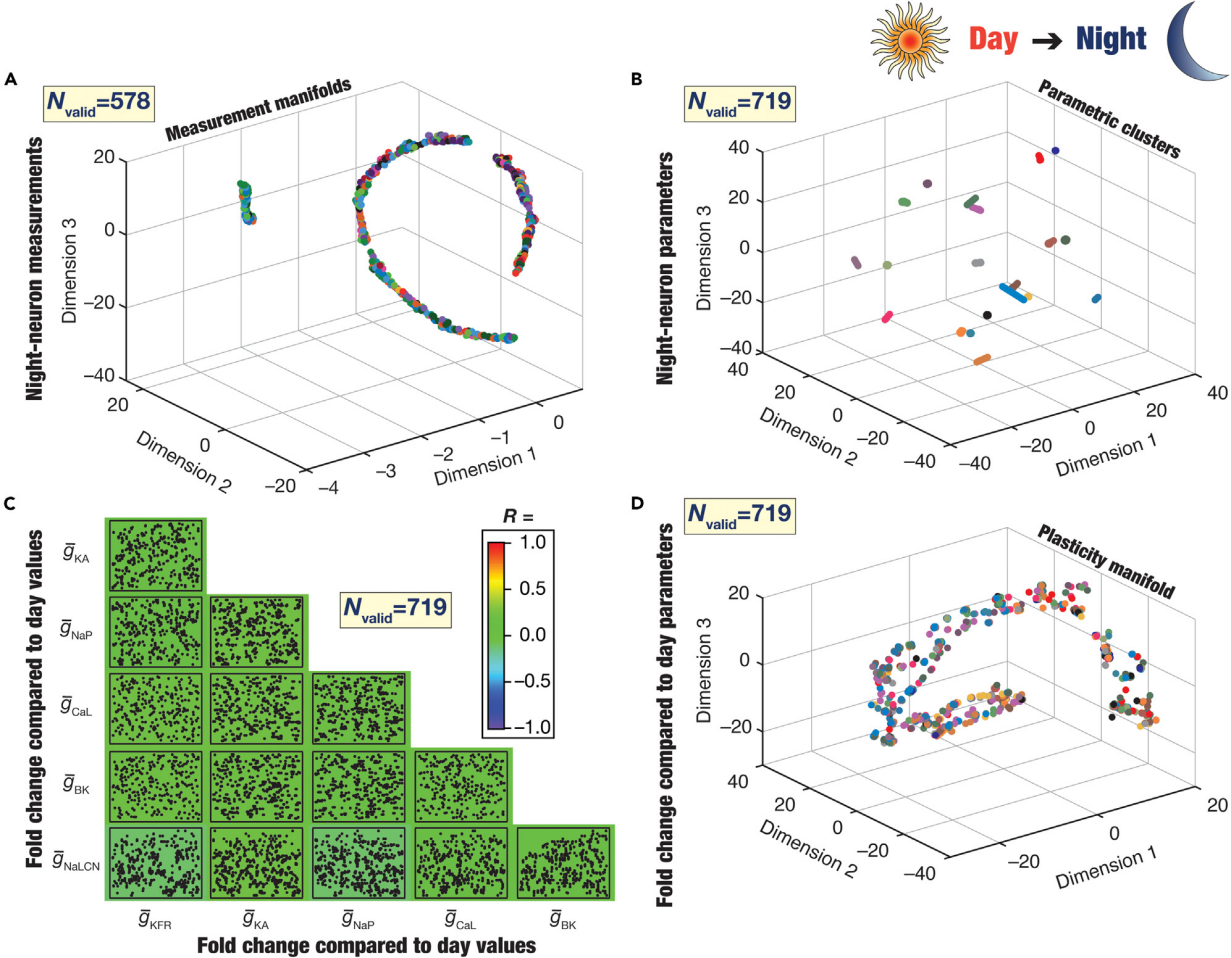
Analyses involving dimensionality reduction of fold-changes in ion-channel conductances involved in day-to-night transitions revealed the existence of plasticity manifolds (A) Representation of the 9 measurements from all 578 intrinsically active night-like neurons on a reduced 3-dimensional space obtained through *t*-SNE. Different colors represent the 20 distinct day-like neurons from which the night-like models were obtained (shown in Figure 4B). The absence of clustering based on the day-like neuron implies that night-like neurons with distinct origin may show similar measurement phenotypes. These analyses were performed on the 578 intrinsically active night-like neurons because the silent neurons did not elicit action potentials making action potential measurements infeasible. (B) Representation of the 13 parameters from all 719 night-like neurons on a reduced 3-dimensional space obtained through *t*-SNE. Different colors represent the 20 distinct day-like neurons from which the night-like models were obtained (shown in Figure 4C). The parameters associated with the night-like neurons formed distinct clusters based on day-like neuron where they transitioned from. (C) Scatterplot matrix showing pairwise relationships between the fold changes in the six ion-channel conductances that underwent plasticity to yield the 719 valid night-like neurons from the 20 day-like neurons. The background color represents the value of the Pearson correlation coefficient between the different fold changes and indicates weak pairwise correlations across all pairs. (D) Representation of the fold changes in the 6 ion-channel conductances that yielded the 719 night-like neurons, with reference to conductance values in their respective day-like counterparts. Fold changes are shown on a reduced 3-dimensional space obtained through *t*-SNE. Different colors represent the 20 distinct day-like neurons from which the night-like models were obtained (ion channel distributions in day-like neurons shown in Figure 4C). Note the absence of clustering based on day-neuron colors. The values spanned a small manifold within the allowed space of all transitions, illustrating a structured regime involving plasticity in different ion channels that governed day-to-night transitions.

How constrained was the measurement space, the parametric space, and the fold changes associated with the valid night-like neurons and the transitions that resulted in these neurons? An ideal way to visualize constraints of a given set of data points in an *N*-dimensional space is to use dimensionality reduction techniques. We applied nonlinear dimensionality reduction techniques on the 9-dimensional measurement space (Figure 6A) and the 13-dimensional parametric space (Figure 6B) associated with the 719 valid night-like neurons. We also assessed pairwise relationships between plasticity in the ion-channel conductances (Figure 6C) as well as a dimensionality reduction analysis on the 6-dimensional plasticity space (Figure 6D) that yielded these valid night-like neurons. The night neurons were color-coded based on the specific 20 day-like neurons from where these neurons transitioned to manifest night-like characteristics (Figures 6A, 6B, 6D).

These analyses provided several critical insights about the circadian oscillations observed in intrinsic electrophysiological properties of SCN neurons. First, these analyses showed that the night-like neurons occupied a low-dimensional manifold in the 9-dimensional measurement space (Figure 6A). These observations demonstrated that the measurement space associated with these neurons, required to match the characteristic night-like properties of SCN neurons, was indeed constrained and did not involve arbitrary measurement combinations. Importantly, the measurement space did not manifest clusters that were defined by the specific day-neuron origin of the night-like neurons (Figure 6A). This implied that the measurement space was not constrained by the specific day-like neuron where the night-like neuron originated, and manifested intermingled distribution in the reduced dimensionality space (Figure 6A). Together, the measurements from night-like neurons were not distinguishable based on the origin day-like neuron and were constrained within a low-dimensional manifold in the measurement space.

Second, turning to the parametric space, we found 20 distinct clusters of parameters governing the 719 valid night-like models, with each cluster related to the 20 distinct day-like neurons where they transitioned from (Figure 6B). This was in striking contrast to the similarity of the measurements observed across these neurons (Figure 6A), and together provided a clear visualization of degeneracy, by showing the disparate parametric combinations that could result in the emergence of similar night-like characteristic functions (Figure 6A and B). It is important to emphasize that despite the clusters observed, not all perturbations (plasticity in the 6-dimensional space) from the original day-like model yielded valid night-like models as a majority were declared invalid. Thus, despite the heterogeneities in the ion-channel expression profile of individual day-like neurons (Figure 4C), the transitioned night-like neurons manifested similar measurements (Figure 6A) but with parameters falling into distinct clusters (dependent on the origin day-like neuron) in the parametric space (Figure 6B).

Finally, we asked whether the plasticity observed was constrained or occupied the entire possible range of the 6-dimensional plasticity space (in Table 3). There were no strong pairwise relationships between plasticity in any pair of channel conductances (Figure 6C), suggesting the absence of correlated plasticity in conductances that resulted in valid night-like models. We performed nonlinear dimensionality reduction analysis on this 6-dimensional plasticity space, to visualize the manifestation of manifolds or clusters in plasticity space (Figure 6D). Strikingly, we found that the fold changes from day-like parameters that resulted in the valid night-like parameters were constrained within a low-dimensional manifold within the plasticity space (Figure 6D). This is surprising because this plasticity manifold was observed despite the heterogeneities in the origin day-like model measurements and parameters (Figure 4C), the heterogeneities in the valid night-like model measurements (Figure S5) and parameters (Figure 5A), the presence of clusters in the night-like parametric space (Figure 6B), and the absence of pairwise relationship between plasticity measurements across these models (Figure 6C). Importantly, there was no clustering based on the origin day-like neuron, and valid transitions were spread throughout the plasticity manifold. Our conclusions on nonlinear dimensionality reduction analyses on the measurement, parametric, and plasticity spaces associated with these night-like neurons were invariant to the specific dimensionality reduction technique employed for the analyses (results for UMAP and PHATE on these datasets are shown in Figure S7).

Together, despite the absence of strong correlations between plasticity in individual conductances (Figure 6C), only a subset of *combination of changes forming a low-dimensional plasticity manifold* was permitted as valid day-to-night transitions, irrespective of the origin day-like models (Figure 6D). These observations implied that the precise magnitudes of plasticity allowed for each of the 6 ion-channel conductances were constrained to specific combinations (Figure 6D) despite widespread heterogeneities in the origin conductance values themselves (Figure 4C).

### Absence of plasticity manifolds in the night-to-day transitions

**Figure 7:**
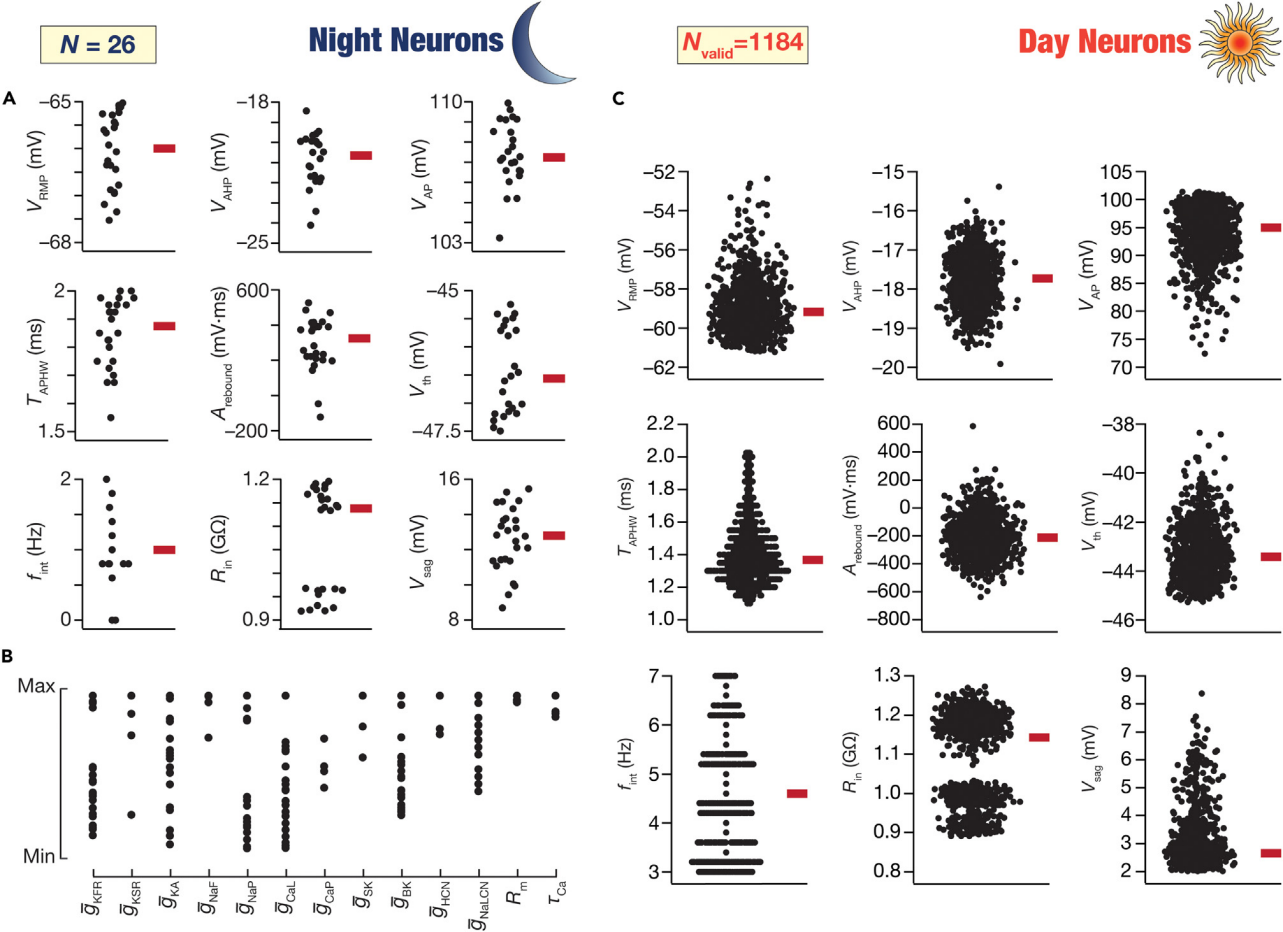
Heterogeneous distribution of measurements from day-like SCN neurons derived through unbiased search of transitions from night-like neurons (A and B) Bee-swarm plots of measurements (A) and parameters (B) of the 26 night-like SCN neuron models that were subjected to the night-to-day transition. The transition was implemented through a modified MPMOSS algorithm that was employed to perform an unbiased search on the plasticity space. The plasticity space accounted for the physiological direction of changes in the six ion channels (Figure 4A) that are known to undergo plasticity during circadian oscillations. The widespread distribution of the measurements and the parameters of the 26 night-like neurons may be noted. (C) Bee-swarm plots of the measurements from 1184 different SCN day-like neurons derived from the 26 night-like neurons. Red bars represent the median values. The validation process for obtaining day-like neurons employed established electrophysiological bounds on each measurement (Table 1).

These day-to-night transitions constitute one-half of the circadian oscillatory cycle. To complete the circadian cycle, we repeated the process of picking heterogeneous population of night-like models, subjecting them to randomized sign-enforced plasticity, and validating them against day-like electrophysiological measurements (Figure 1). Specifically, we picked 26 night-like neurons which manifested heterogeneities in their measurements (Figure 7A) and parameters (Figure 7B). We picked these 26 models specifically from 5 different day-like neurons in the previous half cycle, so that we can track the transitions across the entire cycle. We subjected these 26 models to a sign-enforced stochastic search spanning a 6-dimensional plasticity space involving ion-channel conductances that are known to undergo plasticity during night-to-day transitions (Table 4). Specifically, for each of the 26 night-like neurons, we generated 1000 to 120,000 random samples with a goal of generating at least 20 (max 206 from a night-like neuron) valid night-to-day transitions from each neuron. This process together yielded 1184 valid night-to-day transitions from a total of 273,000 random transitions that were generated, with all neurons that were declared valid showing day-like characteristics (Figure 7C; *cf*. Table 2). Importantly, these 1184 day-like neurons manifested pronounced heterogeneities within the valid measurement ranges (Figures 7C and S8), thus providing us with a heterogeneous population of day-like neurons that have completed one full cycle of circadian oscillations.

**Table 4:** Range of plasticity parameters used in the night-to-day transitions

	Parameter (Unit)	Range of Δg	Relation between $g_{day}$ and $g_{night}$
1	${\bar{g}}_{KFR}$ (mS/cm^2^)	(0,10)	$g_{Day}^{KFR}=g_{Night}^{KFR}\;\left(1+\Delta g\right)$
2	${\bar{g}}_{KA}$ (mS/cm^2^)	(0,10)	$g_{Day}^{KA}=g_{Night}^{KA}\;\left(1+\Delta g\right)$
3	${\bar{g}}_{NaP}$ (mS/cm^2^)	(0,10)	$g_{Day}^{NaP}=g_{Night}^{NaP}\;\left(1+\Delta g\right)$
4	${\bar{g}}_{CaL}$ (mS/cm^2^)	(0,10)	$g_{Day}^{CaL}=g_{Night}^{CaL}\;\left(1+\Delta g\right)$
5	${\bar{g}}_{BK}$ (S/cm^2^)	(0,1)	$g_{Day}^{BK}=g_{Night}^{BK}\;\left(1-\Delta g\right)$
6	${\bar{g}}_{NaLCN}$ (μS/cm^2^)	(0,10)	$g_{Day}^{NaLCN}=g_{Night}^{NaLCN}\;\left(1+\Delta g\right)$

Pairwise measurement correlations (Figure S9) were comparable to those obtained with the original day-like measurements (Figure 4B) with input resistance showing distinct clusters owing to disparate origins (Figures 7C, S9). The parameter ranges for all the 6 ion-channel conductances undergoing plasticity spanned beyond the ranges specified for the generation of the initial set of valid day-like models (Figures S10A–F, S11, *cf.* Table 1). The parameters that underwent plasticity showed weak pairwise correlations (Figure S10G), with large distances between model parameters in the 13-dimensional parametric space (Figure S10H). The distances between these models are relatively smaller (*cf.* Figure 3D) because they all originated from 5 day-like neurons after completion of one full cycle of circadian oscillations with only a subset of 6 parameters changing during these transitions. Origin-dependent clusters were observed in the reduced dimensional measurement and parametric spaces after linear dimensionality reduction analysis (PCA) on these 1184 neurons (Figure S12).

**Figure 8:**
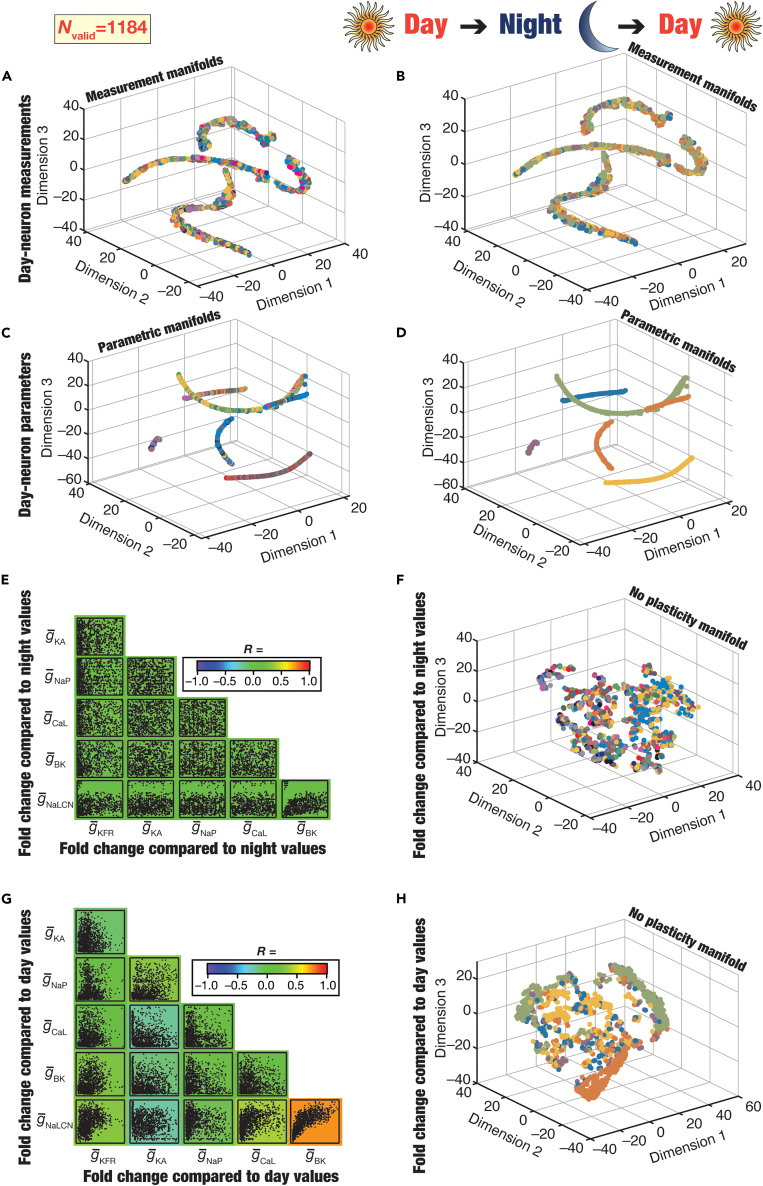
Dimensionality-reduction analyses of fold-changes in ion-channel conductances involved in night-to-day transitions revealed the absence of structured plasticity manifolds (A and B) Representation of the 9 measurements from all 1184 day-like neurons on a reduced 3-dimensional space obtained through *t*-SNE. Different colors represent the 26 distinct night-like neurons (A) from which the day-like models were obtained or the 5 original day-like neurons (B) where the 26 night-like neurons transitioned from. The absence of clustering based on the night-like neuron implies that day-like neurons with distinct origin may show similar measurement phenotypes. (C and D) Representation of the 13 parameters from all 1184 day-like neurons on a reduced 3-dimensional space obtained through *t*-SNE. Different colors represent the 26 distinct night-like neurons (C) from which the day-like models were obtained or the 5 original day-like neurons (D) where the 26 night-like neurons transitioned from. Day-like neurons formed distinct clusters, which were fewer than the number of distinct night neurons. (E) Scatterplot matrix showing pairwise relationships between the fold changes in the six ion-channel conductances that underwent plasticity to yield the 1184 valid day-like neurons from the 26 night-like neurons. The background color represents the value of the Pearson correlation coefficient between the different fold changes and indicates weak pairwise correlations across all pairs. (F) Representation of the fold changes in the 6 ion-channel conductances that yielded the 1184 day-like neurons, with reference to conductance values in their respective night-like counterparts. Fold changes are shown on a reduced 3-dimensional space obtained through *t*-SNE. Different colors represent the 26 distinct night-like neurons from which the day-like models were obtained (ion channel distributions in night-like neurons shown in Figure 7B). Note the absence of clustering based on night-neuron colors. (G–H) Same as (C and D), but the fold-change analyses were performed with reference to the 5 original day-like neurons where the 26 night-like neurons were derived from, and eventually led to the 1184 day-like neurons.

We performed nonlinear dimensionality reduction analyses on the 9-dimensional measurement space (Figure 8A and 8B), the 13-dimensional parametric space (Figures 8C and 8D), and the 6-dimensional plasticity space (Figure 8F) to visualize the global structure of the valid day-like neurons and the associated transitions. We found measurement manifolds in day-like neurons showing that the day-like SCN measurements were also constrained within a small subset of the 9-dimensional measurement space (Figures 8A and 8B). Importantly, day-like models that transitioned from different night-like neurons did not manifest separate clusters but were intermingled within this low-dimensional measurement manifold showing similarity of measurements irrespective of origin neurons (Figure 8A and 8B). On the other hand, the parametric space clustered within distinct manifolds, with each cluster specifically related to the original day-like neuron where they originated from (Figures 8C and 8D). It may be noted that the extent of each cluster/manifold (associated with single origin neurons) in the reduced parametric space in Figure 6B was smaller compared to that in Figure 8D. Similar functional outcomes obtained from disparate combinations of underlying parameters point to the expression of ion-channel degeneracy in these day-like neurons (which have undergone one full circadian oscillatory cycle) as well. We did not observe strong pairwise correlation between the plasticity across different ion channels that underwent plasticity during the night-to-day transitions (Figure 8E). In striking contrast to the plasticity manifolds observed with day-to-night transitions (Figure 6D), we did not observe a low-dimensional manifold in the 6-dimensional plasticity space associated with the night-to-day transitions (Figure 8F).

As these neurons have undergone one full cycle of circadian oscillation, we assessed the cumulative plasticity undergone by these neurons through this one full cycle of oscillation. We found that there were no strong pairwise correlations between individual pairs of conductance changes (Figure 8G) when fold changes in the 6 conductances of the 1184 days-neurons were compared to their 5 day-like originators. In addition, there was no plasticity manifold observed when these fold changes were subjected to dimensionality reduction analyses, with intermingling spanning different originators across a distributed global structure (Figure 8H). Our conclusions on nonlinear dimensionality reduction analyses on the measurement, parametric, and plasticity spaces associated with these day-like neurons were invariant to the specific dimensionality reduction technique employed for the analyses (results for UMAP and PHATE on these datasets are shown in Figure S13). Our conclusions about the measurement, parametric, and plasticity spaces held even when we restricted dimensionality reduction analyses to day-like neurons that transitioned from 5 distinct night-like neurons, which in turn transitioned from 5 distinct day-like neurons (Figure S14).

Although the sign of plasticity in each individual ion channel was opposite for day-to-night and night-to-day transitions, we did not find the cumulative plasticity in individual ion channels to be zero (Figure 8G and H). In other words, ion-channel conductances did not revert to their original values in individual models in the process of returning to day-like measurements. There was considerable heterogeneity in plasticity of individual ion channels across the full circadian cycle, even for models derived from a single day-like model (Figure 8H), thus pointing to the expression of plasticity degeneracy in circadian transitions. As mentioned earlier, the conductance values of the transitioned day-like models exceeded the ranges set for the original day-like models (Figure S11), providing additional evidence that there was no restoration of the original ion-channel expression profiles. The ability to revert to precise day-like physiology despite not reinstating the precise ion-channel expression profiles of the original day-like neuron has critical implications for circadian physiology. These analyses provide elegant illustrations of the co-expression of ion-channel degeneracy and plasticity degeneracy in SCN neurons, which together form the substrate for robust and precise functional transitions through multiple possible routes in a heterogeneous neuronal population.

**Figure 9:**
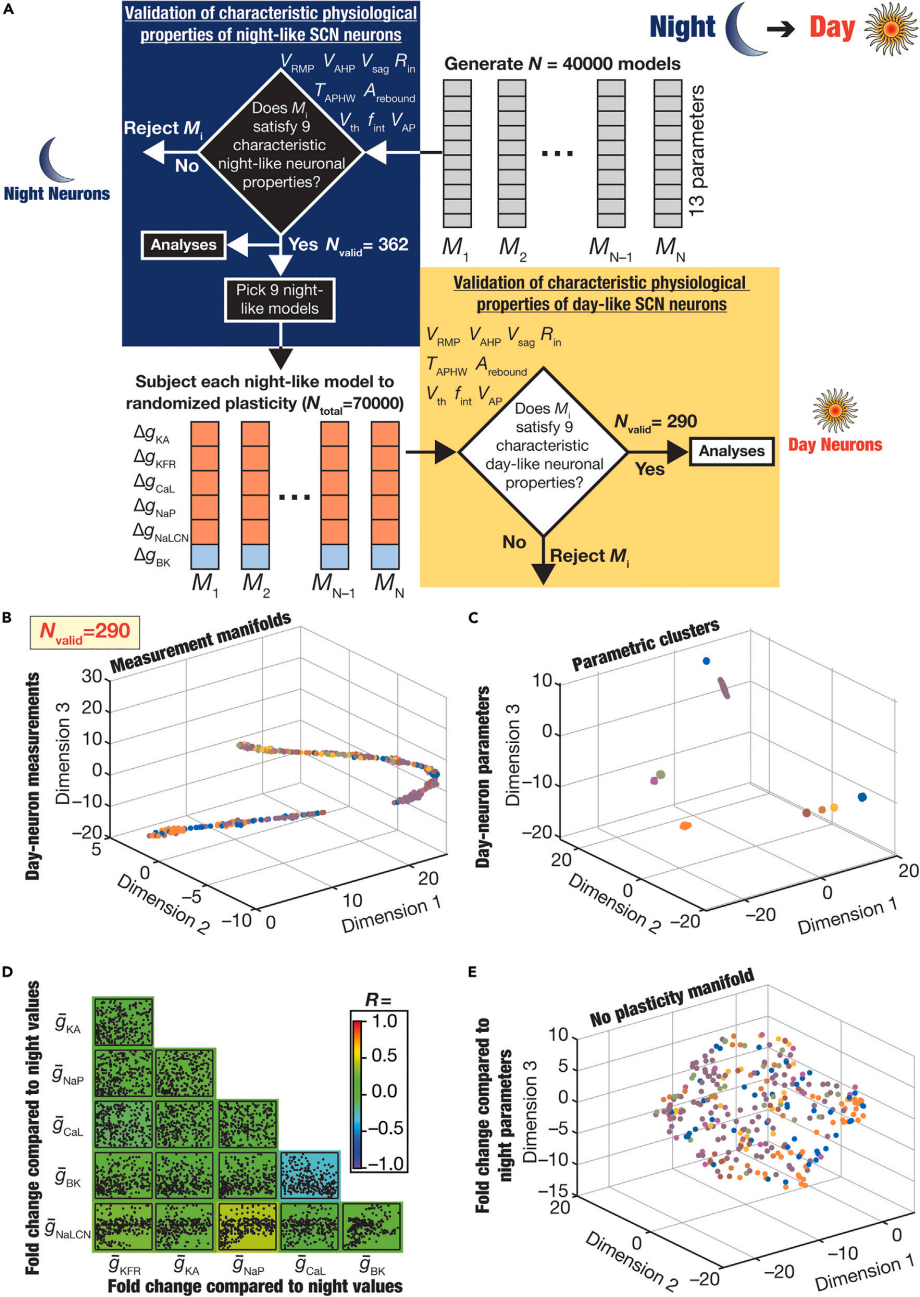
Dimensionality-reduction analyses of fold-changes in ion-channel conductances involved in night-to-day transitions revealed the absence of plasticity manifolds from a *de novo* population of night-like neurons (A) Flowchart illustrating the overall methodological plan for assessing ion-channel degeneracy and plasticity manifolds in circadian oscillations in SCN neurons with the cycle starting at a heterogeneous population of night-like neurons. *Left,* The first set of night-like neurons were generated by a *de novo* unbiased search involving 13 different parameters involving 40000 neuron models. Of these, 362 were found to show valid night-like physiological properties. As a second step, 9 of these 362 night-like models were picked and subjected to night-to-day transitions that involved plasticity in six different ion channels in electrophysiologically determined directions (red implies increase, blue implies reduction). *Right,* Models subjected to randomized plasticity (*N*_total_ = 70,000 total random transitions) were validated with day-like measurements from SCN neurons, and 290 models derived from the 9 day-like models were found to be valid. The specific combinations of ion-channel plasticity (from respective night-like neurons) that resulted in valid day-like models were subjected to dimensionality reduction analysis to determine the presence of structured plasticity manifolds in the night-to-day transitions. (B) Representation of the 9 measurements from all 290 day-like neurons on a reduced 3-dimensional space obtained through *t*-SNE. Different colors represent the 9 distinct night-like neurons from which the day-like models were obtained (shown in Figure S15). The absence of clustering based on the night-like neuron implies that day-like neurons with distinct origin showed similar measurement phenotypes. (C) Representation of the 13 parameters from all 290 night-like neurons on a reduced 3-dimensional space obtained through *t*-SNE. Different colors represent the 9 distinct night-like neurons from which the day-like models were obtained (shown in Figure S15). The parameters associated with the day-like neurons formed distinct clusters based on night-like neuron where they transitioned from. (D) Scatterplot matrix showing pairwise relationships between the fold changes in the six ion-channel conductances that underwent plasticity to yield the 290 valid day-like neurons from the 9 night-like neurons. The background color represents the value of the Pearson correlation coefficient between the different fold changes and indicates weak pairwise correlations across all pairs. (E) Representation of the fold changes in the 6 ion-channel conductances that yielded the 290 day-like neurons, with reference to conductance values in their respective night-like counterparts. Fold changes are shown on a reduced 3-dimensional space obtained through *t*-SNE. Different colors represent the 9 distinct night-like neurons from which the day-like models were obtained (ion channel distributions in night-like neurons shown in Figure S15). Note the absence of clustering based on night-neuron colors and the absence of plasticity manifolds defining the night-to-day transitions from a *de novo* population of night neurons.

Could the manifestation of plasticity manifolds in day-to-night, but not in night-to-day, transitions be an artifact of our methodology (in Figure 1) where we first generate day neurons *de novo*? Could it be that the first transition from *de novo* neuronal population is restricted to a manifold while the second transition does not manifest a manifold? To address these questions, we generated a *de novo* population of night-like SCN neurons (Figure 9A) and subjected those to night-to-day transitions to explore the manifestation of plasticity manifolds. Specifically, we generated 40,000 random models in the parametric ranges from Table 1 and subjected them to validation against night-like measurements (Table 2). We found 362 valid night-like neurons, of which we subjected 9 heterogeneous neurons (Figures S15A and S15B) to night-to-day transitions (Figure 9A). We explored the night-to-day plasticity space (defined by Table 4) by generating 70,000 randomized transitions. We subjected each of these transitioned models to a validation process involving day-like physiological properties (Table 2) and found 290 valid day-like models from the 9 night-like neurons (Figures 9A, S15C). These 290 models were endowed with pronounced heterogeneities in their measurements (Figure S15C) but fell within a measurement manifold in a reduced dimensional space obtained with *t*-SNE (Figure 9B). The parametric space associated with these 290 day-like neurons manifested clusters based on the specific night-like neuron they originated from (Figure 9C). There were no strong pairwise correlations between the plasticity that the 6 different ion channel densities underwent (Figure 9D), thus ruling out the need for a single mechanistic basis for plasticity in all ion channels toward achieving valid transitions. There was also a striking lack of the manifestation of a plasticity manifold in the reduced dimensionality plasticity space obtained with *t*-SNE on these valid night-to-day transitions (Figure 9E). These analyses (Figures 9, S15) confirmed the absence of a plasticity manifold in night-to-day transitions, even when a *de novo* heterogeneous population of night neurons was subjected to transitions that yielded valid day-like neurons (compare Figure 9 with *de novo* neurons with Figure 8 obtained with transitioned neurons).

Turning to parametric spaces, the distinct clusters/manifolds observed in the reduced parametric spaces across different populations (Figures 6*B*, 8*C–D*, 9*C*, S7*C–D*, S13*C–D*, S14*B*) are simply reflections of the origin neurons. However, the extent of the cluster/manifold associated with a specific ancestor was small in Figure 6B, but the extent of the cluster was larger in Figure 8D. Our analyses with *de novo* night neurons undergoing a single day-to-night transition (Figure 9) demonstrate that these differences (between Figure 6B and Fig. 8D) are not attributable to distinctions between day-like and night-like neurons. Instead, these are differences between a neuronal population that underwent one (day-to-night) transition from 20 distinct ancestors (Figure 6B) vs. a population that underwent two (day-to-night and night-to-day) transitions from 5 distinct ancestors (Figure 8D). We arrived at these conclusions by noting the extents of the clusters/manifolds in the reduced parametric space of day-like neurons that were obtained from a single transition from 9 distinct ancestors (Figure 9C). The extents of the distinct clusters/manifolds in these day-like neurons obtained after a single transition (Figure 9C) were comparable to night-like neurons after a single transition (Figure 6*B*) rather than day-like neurons after two transitions (Figure 8D). Thus, the extent of the individual clusters/manifolds in the reduced parametric space (Figures 6B, 8C, 8D, 9C, S7C, S7D, S13C, S13D, S14B) was not dependent on day-like and night-like characteristics but was instead dependent on number of transitions (which enhances heterogeneity) and the number of distinct ancestors (larger parametric space explored with more ancestors).

Together, these analyses suggest greater flexibility in night-to-day transitions (Figures 8, 9, S13–S15) compared to their day-to-night counterparts (Figures 6, S7), with plasticity manifolds manifesting for day-to-night transitions, but not for night-to-day transitions.

## Discussion

The overall experimental design involving a heterogeneous population of biophysically realistic SCN model neurons undergoing one full cycle of circadian oscillations (Figure 1) yielded several important insights about SCN neuronal physiology. First, our analyses demonstrated the expression of ion-channel degeneracy, whereby disparate ion-channel combinations yielded SCN neurons with characteristic day- or night-like physiological properties. Second, efficacious circadian transitions in all intrinsic physiological measurements were observed despite the pronounced parametric heterogeneities that governed the day- or night-like neurons. Importantly, successful physiological transitions were feasible despite strong physiological restrictions on the identity and sign of plasticity observed in the different conductances as well as the heterogeneous expression of these conductances across different models. Third, our results unveiled plasticity degeneracy, whereby disparate combinations of sign-enforced plasticity in the 6 identified ion-channel conductances yielded valid day-to-night or night-to-day transitions in the intrinsic properties of SCN neurons. Plasticity degeneracy was observed even within a single neuron, where disparate combinations of parametric changes could yield valid transitions in intrinsic properties from the same origin neuron. These observations imply that at any specific phase of circadian oscillations spanning multiple cycles, individual SCN neurons should manifest pronounced parametric variability despite physiological similarity. Finally, we report the expression of plasticity manifolds, that constrain plasticity within a low-dimensional manifold within the higher dimensional plasticity space involving all possible changes, in the emergence of circadian transitions.

### Ion-channel degeneracy in the manifestation and circadian transitions of intrinsic properties: Implications for heterogeneities in SCN neuronal population

The question of how neurons in the SCN undergo circadian oscillations despite widespread heterogeneities in their biophysical composition and intrinsic properties is fundamental to understanding the precise cell-autonomous circadian changes in intrinsic properties.^1^^,^^6^^,^^7^^,^^21^ An important methodological distinction in our study with reference to prior modeling studies on the biophysical basis of SCN oscillations in different model systems^29^^,^^30^^,^^31^^,^^32^ is the ability to explicitly account for each heterogeneity (in biophysical and intrinsic properties) in an unbiased manner. Incorporation of these heterogeneities provided important insights involving ion-channel degeneracy, plasticity degeneracy, and plasticity manifolds to be important contributors to the robustness of cell-autonomous oscillations of intrinsic properties of SCN neurons. Specifically, our analyses involving a biophysically and physiologically constrained heterogeneous population of SCN neuronal models provide an elegant solution to this question from within the degeneracy framework.^23^^,^^24^^,^^25^^,^^26^^,^^33^^,^^34^^,^^35^^,^^36^^,^^37^ Our analyses show that parametric heterogeneities need not be an impediment to the concomitant emergence of 9 different (Table 2) characteristic intrinsic properties of day-like (Figures 2, 3, 7, S3, S8–S11, S15) or night-like (Figures 4, 5, S8, and S11) SCN neurons. The ability of disparate ion-channel combinations to yield similar characteristic physiology, with these ion channels constrained by respective biophysical measurements, shows that the composition of all SCN neurons even in the same animal need not be same for them to manifest similar physiological characteristics.

In addition, although there were strong constraints on the identity of the ion channel and the direction in which they are permitted to change, our analyses show that disparate combinations of ion-channel plasticity could yield valid day-to-night (Figure 6C) or night-to-day (Figure 8E) transitions. These observations have several important implications. First, SCN neurons are not tightly constrained by a unique set of plasticity in the underlying channels. They are endowed with considerable freedom in choosing among several sets of transitions to make a valid physiological transition during circadian cycles. Second, if one were to follow the ion-channel composition of a single SCN neuron at a specific phase across several circadian cycles, there could be pronounced variability in the biophysical parameters across different cycles (Figure S11) despite the physiological measurements being characteristically similar (Figure S8). Specifically, let us say that we measure the ion-channel composition and the physiological measurements of a single SCN neuron at the same time of the day for several days. Our analyses demonstrate that while the physiological measurements across days could be identical or similar, the parametric combinations in this single neuron that yielded these physiological outcomes need not be similar. In other words, upon undergoing a full cycle, the neuron need not return to the original parametric values despite constraints on the intrinsic physiological measurements to return to similar values. We note that the possibility of pronounced parametric variability despite measurement similarity across oscillatory cycles in SCN neurons constitutes an important experimentally testable prediction within the degeneracy framework.

Third, these observations imply that the plasticity required by the same neuron to implement a valid transition (day-to-night or night-to-day) should be very different across cycles. If the parametric composition of the individual neuron changes across cycles, it stands to reason that the same magnitudes of changes that were employed in the previous cycle might not yield a valid physiological transition in this cycle. Plasticity degeneracy provides an elegant framework for achieving valid transitions not just across neurons in a population manifesting parametric heterogeneities^38^^,^^39^^,^^40^ but also to individual neurons transitioning across cycles through different combinations of ion-channel plasticity. Plasticity degeneracy implies that the set of ion channels and mechanisms mediating circadian oscillations could be very different in adjacent SCN neurons as well as in different cycles of the same neuron. Together, these observations emphasize the context dependence of plasticity required to achieve valid transitions, in a manner that is tied to the current composition of the neuron undergoing the transition. Such context-dependent transitions could be further explored using computational analyses involving continual transitions as well as multi-cycle, multi-scale experimental measurements from SCN neuron cultures.

### Plasticity manifolds involving ion channels mediating circadian oscillations of intrinsic properties

Experimental lines of evidence show that SCN neurons undergo transitions from a day-like to a night-like state through concurrent changes in channel densities only in a subset of ion channels that are expressed by them.^11^^,^^12^^,^^13^^,^^14^^,^^15^^,^^16^^,^^17^ It is plausible that structured rules that govern the changes in channel densities might permit only certain combinations of changes to lead to transitions. Under such a scenario, the permitted plasticity combinations would form a lower dimensional manifold in the space of allowed changes, which is termed a plasticity manifold.^28^^,^^41^

We report the manifestation of plasticity manifolds in case of the day-to-night transition but not the night-to-day transition. This was visualized using nonlinear dimensionality reduction analyses, which have been widely used in the neural manifolds literature,^42^ but have not been applied to analyzing manifolds in plasticity space.^28^ Our analyses demonstrate the existence of such plasticity manifolds, pointing to the presence of strong constraints on the magnitudes of sign-enforced plasticity on the ion channels. These constraints in plasticity are tightly intercoupled to several mechanisms and measurements, including the specific physiological goal(s) to be achieved, the current state of the system, the set of components that are allowed to undergo plasticity, the direction of permitted changes in the plastic component, and structure in the signaling mechanisms that govern simultaneous plasticity in the components.^28^ Our analyses unveiled plasticity manifolds in day-to-night transitions despite the absence of specific signaling mechanisms that governed the concomitant changes. Our search was random and unbiased across the entire permitted plasticity space and did not impose constraints on the coupling between the plasticity in the different ion channels. Despite such independence in individual ion-channel plasticity, and despite heterogeneities in the expression of the 6 ion channels in the origin neurons, we found the manifestation of plasticity manifold in day-to-night transitions, but not in night-to-day transitions.

These observations point to a scenario where constraints at a higher scale (transitions in cellular physiological measurements) imposed specific structure at a lower scale (specific amount of plasticity in individual ion-channel conductances). A critical conclusion is that these constraints allow for a multiplicity of possible routes which fall into a structured plasticity manifold, rather than one single transition route that would have curtailed flexibility in ion-channel conductance values. A simple plasticity manifold would be a scenario where plasticity of all these ion channels are co-regulated by a single mechanism, therefore resulting in strong pairwise correlations across all observed changes.^24^^,^^43^^,^^44^^,^^45^^,^^46^ However, our analyses show the absence of such strong pairwise correlations (Figures 6C, 8E), instead showing the manifestation of a low-dimensional plasticity manifold^28^ after nonlinear transformation of the plasticity space for day-to-night transitions (Figure 6D). Experimental analyses could be employed to assess these predictions, and computational analyses involving the TTFL could be employed to further address the structure of the plasticity manifold that results in circadian transitions.

The manifestation of a plasticity manifold in day-to-night transition but not in the night-to-day transitions emphasizes the asymmetry in the mechanisms underlying the bidirectionality of physiological transitions. Although there are lines of evidence for asymmetry on the impact of bidirectional changes in other contexts,^47^^,^^48^ our analyses suggest stringent constraints on day-to-night transitions compared to their night-to-day counterparts. The differential flexibilities for the two transitions emanate from the specific physiological targets (Table 2) and the permitted plasticity space involving directions of the subset of ion channels (Tables 3 vs. 4). Our analyses did not account for the specific signaling components involved in plasticity of each of these ion channels or interactions among the various signaling mechanisms that yield simultaneous plasticity in these channels. The incorporation of signaling constraints (and heterogeneities therein) could place additional constraints on the plasticity profiles observed with day-to-night and night-to-day transitions. Future experimental and computational studies could examine the existence as well the mechanistic bases behind such differential constraints in the two transitions.

In summary, our analyses unveil an elegant substrate, involving a synthesis of the degeneracy and the plasticity manifolds frameworks, to implement stable circadian oscillations in a heterogeneous population of SCN neurons. Within this framework, heterogeneities provide a substrate for realizing degeneracy and could emerge from the multiple plasticity routes that can yield valid transitions. The manifestation of plasticity manifolds provides a structure that avoids instability by constraining concomitant plasticity to specific combinations of changes that need not necessarily involve correlated changes in all ion channels. These analyses also argue for the SCN as an ideal system to address conceptual and mechanistic questions on the synergy between heterogeneities, long-term plasticity manifolds, and degeneracy in coupled functional emergence *across multiple scales*. The argument follows from the inherent heterogeneities in SCN neuronal properties and molecular mechanisms as well as the precise long-duration cycles in characteristic measurements spanning several scales. Our analyses emphasize the critical need to account for neural-circuit heterogeneities in studying circadian oscillations and provide an overarching framework to understand the intricately connected multi-scale circadian oscillations in the heterogeneous SCN under physiological and pathological conditions.

### Limitations of the study

Our analyses unveiled important insights about how a heterogeneous population of SCN neurons could manifest characteristic properties and undergo signature physiological transitions across a circadian cycle. However, there are important model considerations that need to be addressed in future studies. First, our analyses are based on a simple, single-compartmental conductance-based model of an SCN neuron and did not place constraints on evoked action potentials in day-like and night-like neuronal models. Our analyses were limited by the lack of detailed sub- and supra-threshold electrophysiological measurements across the somato-dendritic arbor that is essential for constraining models. Future studies could look to incorporate additional structural heterogeneities, as well as heterogeneities in dendritic ion channel distributions, in a morphologically realistic model population that manifests characteristic somato-dendritic properties. Such computational analyses have to be accompanied by morphological and electrophysiological measurements of SCN neurons across the circadian cycle. Electrophysiological measurements should include subthreshold measurements and spontaneous as well as evoked (through synaptic stimulation or pulse current injections) action potential measurements across the somato-dendritic structures. Models constrained by these experimental measurements could then be employed to probe questions about the impact of structural and biophysical heterogeneities on characteristic physiological properties and their signature circadian transitions.

Second, circadian oscillations in the SCN are studied across multiple scales: the cell-autonomous SCN, the molecular mechanisms, and signaling networks underlying continual circadian transitions involving the TTFL in SCN cells, the SCN as a cellular network and, finally, the SCN as circadian orchestrator.^1^^,^^6^^,^^7^^,^^11^^,^^15^^,^^18^^,^^20^^,^^21^ Our study focused on the ionic basis of cell-autonomous changes in neural excitability, specifically looking at two distinct timepoints assigning them as representative of day and night neurons. The insights here about plasticity manifolds and ion-channel degeneracy could be employed to understand the molecular mechanisms and the role of TTFL in regulating these ion channels in the heterogeneous population of SCN cells. As one of the physiological goals is to regulate the firing rate of the neurons over the day-night cycles, these questions could be addressed from the perspective of how these goals are continually achieved while accounting for heterogeneities not just in the ion-channel composition but also in the signaling components associated with the TTFL.

Importantly, a logical next step would be to assess the impact of ion-channel degeneracy and plasticity manifolds in individual neurons on synchronization of cellular oscillations throughout a heterogeneous SCN network. The converse, on how connectivity and synchronization across different neurons could affect the plasticity manifolds that govern the circadian transitions, also constitutes an important question in terms of how the network inputs define ion-channel degeneracy and plasticity manifolds in individual neurons. Such analyses would also provide insights about potential asymmetries between the day-to-night and the night-to-day transitions and about our prediction on differential expression of plasticity manifolds across these two transitions. It is essential that future studies incorporate neural-circuit heterogeneities in assessing SCN networks toward exploring the manifestation of ion-channel degeneracy as well as plasticity manifolds in the heterogeneous network context as opposed to the single-neuron context explored here.

Third, while the cell-autonomous transitions in intrinsic properties and network synchrony through connections form one layer of complexity, an additional layer of complexity arises from the expression of several neuromodulators that influence action potential activity and rhythmicity in the SCN. These neuromodulators regulate the synchronization properties, the baseline excitability, and the plasticity of the circadian neural code with multi-scale impact of SCN physiology. In addition to accounting for heterogeneities in ion channels, calcium regulatory mechanisms, signaling cascades, neurotransmitters and their receptors, and network connectivity, SCN analyses should include heterogeneities in neuromodulatory receptor expression across SCN neurons. Future studies could investigate the impact of multi-scale heterogeneity (spanning molecular, cellular, and network scales) and explore the expression of degeneracy and plasticity manifolds across different scales while accounting for neuromodulation and light entrainment of the SCN network.^1^^,^^7^^,^^21^^,^^28^^,^^32^^,^^49^^,^^50^ The SCN is an ideal system to address questions on long-term plasticity manifolds and degeneracy across scales, using both experimental and computational techniques, given the well-defined function and the tight coupling observed in the physiology and mechanisms across scales. Strong experimentally constrained multi-scale mechanistic models of the SCN can provide deeper insights into how heterogeneities, degeneracy, and plasticity manifolds across different scales interact to provide precise physiology and state-dependent transitions during circadian oscillations.^28^^,^^51^

Finally, there are reports of changes to ion-channel properties and intrinsic properties spanning different neuronal subtypes under several pathophysiological and altered behavioral conditions.^52^^,^^53^^,^^54^^,^^55^^,^^56^^,^^57^^,^^58^^,^^59^ Thus, future studies should explore the impact of altered channel properties/densities under different behavioral and pathological conditions on specific ion channels and the physiology of SCN neurons, as well as the impact on transitions and plasticity manifolds associated with them. Such studies might be performed by systematically removing ion channels from the models using virtual-knockout model analyses^38^^,^^39^^,^^60^^,^^61^^,^^62^^,^^63^^,^^64^^,^^65^ as well as by examining the electrophysiological properties of the SCN channels and neurons spanning the circadian cycle. These analyses could yield insights into the relative contribution of individual channels to circadian oscillations and unveil the impact of ion channelopathies on circadian oscillations across neurological disorders.

## Author contributions

H.N. and R.N. designed experiments; H.N. performed experiments; H.N. analyzed data; H.N. and R.N. wrote the paper.

## STAR★Methods

### Key resources table

**Table:** 

REAGENT or RESOURCE	SOURCE	IDENTIFIER
**Software and algorithms**		

NEURON	(Hines and Carnevale, 2006)^66^	https://www.neuron.yale.edu
Igor Pro	Proprietary software	http://wavemetrics.com
MATLAB	Proprietary software	https://www.mathworks.com
Simulating SCN neurons	This paper	Supplementary ZIP file Code.zip

### Resource availability

#### Lead contact

Further information and requests for resources and reagents should be directed to and will be fulfilled by the Lead Contact, Rishikesh Narayanan (rishi@iisc.ac.in).

#### Materials availability

This study did not generate new unique reagents.

### Method details

The overall methodological procedure employed in this study is provided in Figure 1. We employed a single-compartmental cylindrical model of an SCN neuron (Figure S1A; diameter, d = 22.3 μm; length, L = 22.3 μm). Passive properties were incorporated as an RC circuit, with a specific membrane resistance $R_{m}$ and a specific membrane capacitance $C_{m}$. $C_{m}$ was set at 1 μF/cm^2^ and $R_{m}$ was chosen to be in the range of 20–40 kΩ cm^2^ to obtain a membrane time constant (= $R_{m}C_{m}$) of 20–40 ms^66^ The leak conductance $g_{L}$, governing the leak current $i_{L}$, was defined as the reciprocal of $R_{m}$. The geometry of the neuron was adjusted to obtain a passive input resistance ($R_{m}/\pi \;d\;L$) in the range of 1.28–2.56 GΩ, matching with electrophysiological measurements from day-like SCN neurons.^67^^,^^68^ The model comprised 11 active channels: fast sodium (NaF), persistent sodium (NaP), sodium leak (NaLCN), fast delayed rectifier potassium (KFR), slow delayed rectifier potassium (KSR), *A*-type potassium (KA), small-conductance Ca^2+^-activated potassium (SK), big-conductance Ca^2+^-activated potassium (BK), *P*-type calcium (CaP), *L*-type calcium (CaL), and the hyperpolarization-activated cyclic-nucleotide gated nonspecific cationic (HCN) channels. The voltage equation that governed the model neuron was thus given by:

$$C_{m}\frac{dV}{dt}=-\left(i_{L}+i_{NaF}+i_{NaP}+i_{NaLCN}+i_{KFR}+i_{KSR}+i_{KA}+i_{SK}+i_{BK}+i_{CaP}+i_{CaL}+i_{h}\right)$$

#### Ion-channel gating properties and kinetics

The channel kinetics and voltage-dependencies for KFR and KA were adopted from Bouskila et al.^69^; for SK and CaP from Huang et al.^70^; for NaF from Sim et al.^71^; for NaP from Paul et al.^15^; for NaLCN from Chua et al.^72^; for BK from Cui et al.^73^; for HCN from de Jeu et al.^74^; for CaL from Diekman et al.^29^ and KSR from Itri et al*.*^12^

All channel models were based on the Hodgkin-Huxley formulation.^75^ The sodium, potassium and HCN channels followed the Ohmic formulation, while the calcium channels followed the Goldman-Hodgkin-Katz (GHK) formulation.^76^^,^^77^ The reversal potentials for Na^+^, K^+^, and HCN were set at +45, −97 and −30 mV respectively.^71^^,^^74^ The evolution of cytosolic calcium concentration ${\left[Ca\right]}_{i}$ was dependent on the current through the voltage-gated calcium channels and involved a first-order decay with a decay time constant in the range of 1750–2240 ms^29^:

$$\frac{d{\left[Ca\right]}_{i}}{dt}=-\frac{10000\;I_{Ca}}{36\;D\;F}+\frac{{\left[Ca\right]}_{\infty }-{\left[Ca\right]}_{i}}{\tau_{Ca}}$$

where *F* represents Faraday’s constant, $\tau_{Ca}$ represents the decay time constant of calcium in SCN neurons, *D* was the depth of the shell into which calcium influx occurs (*D =* 0.1 μm), and ${\left[Ca\right]}_{\infty }$ was the steady state value of ${\left[Ca\right]}_{i}$ set at 50 nM.

Channel models were directly adopted from previous studies when available. In case channel models were not available, they were defined to match respective electrophysiological measurements. The channels were described by either one or two gating particles, with each particle following the first-order kinetics:

$$\frac{dm}{dt}=\frac{m_{\infty }-m}{\tau_{m}}$$

where $m_{\infty }$ and $\tau_{m}$ defined the steady-state value and the time constant of the state variable governing the gating particle, respectively. Channel gating and kinetics were adjusted to account for temperature dependence as well. The details of the gating properties as well as kinetics for each channel are as follows (Figure S1).

### Fast delayed rectifier potassium channel (KFR)

The model for KFR channel was obtained by fitting the corresponding electrophysiological data,^69^ and the current through this channel was as follows (Figure S1B):

$$i_{KFR}={\bar{g}}_{KFR}n^{4}\;\left(V-E_{K}\right)$$

The activation gating particle was governed by:

$$n_{\infty }=\frac{1}{{\left(1+\exp\left(\frac{V-14}{-17}\right)\right)}^{0.25}}$$

$$\tau_{n}=\frac{1}{\alpha +\beta }$$

$$\alpha \left(ms^{-1}\right)=0.16\;\exp\left(\frac{V+20}{-49}\right)$$

$$\beta \left(ms^{-1}\right)=0.11\;\exp\left(\frac{V+20}{30}\right)$$

### Fast sodium channel (NaF)

The model for NaF was adopted from the sodium channel model in Sim et al.*,*^71^ and the current through this channel was (Figure S1C):

$$i_{NaF}={\bar{g}}_{NaF}m^{3}\;h\left(V-E_{Na}\right)$$

The activation gating variable was defined by:

$$m_{\infty }=\frac{1}{1+\exp\left(\frac{V+35.2}{–8}\right)}$$

$$\tau_{m}=\exp\left(\frac{V+286}{-160}\right)$$

The inactivation gating particle was defined by:

$$h_{\infty }=\frac{1}{1+\exp\left(\frac{V+62}{4}\right)}$$

$$\tau_{h}=0.51+\exp\left(\frac{V+26.6}{-4}\right)$$

### Persistent sodium channel (NaP)

The model for NaP was adopted from the persistent sodium channel kinetics provided by Paul et al*.*^15^ The current through the channel was given by (Figure S1D):

$$i_{NaP}={\bar{g}}_{NaP}\;p\;\left(V-E_{Na}\right)$$

The activation gating variable was governed by:

$$p_{\infty }=\frac{1}{{\left(1+\exp\left(\frac{V+25}{-7.4}\right)\right)}^{1.5}}$$

$$\tau_{p}=100$$

### P-type calcium channel (CaP)

The model for CaP (Figure S1E) was adapted from Huang et al*.*^70^ The current through this channel followed GHK conventions. The default extracellular and cytosolic calcium concentrations were set at 2 mM and 100 nM respectively.

$$i_{CaP}={\bar{g}}_{CaP}\;p^{2}\;GHK\left(V,{\left[Ca\right]}_{i},{\left[Ca\right]}_{o}\right)$$

The activation gating variable was governed by (Figure S1E):

$$p_{\infty }=\frac{1}{{\left(1+\exp\left(\frac{V+8}{-5.7}\right)\right)}^{0.5}}$$

$$\tau_{p}=\frac{1.3}{\alpha +\beta }$$

$$\alpha =0.1967\frac{V-37.88}{1-\exp\left(\frac{V-37.88}{-10}\right)}$$

$$\beta =0.046\;\exp\left(\frac{V-18}{-20.73}\right)$$

### A-type potassium channel (KA)

The model for KA was obtained by fitting the corresponding electrophysiological data.^69^ The current through the channel was defined as follows (Figure S1F):

$$i_{KA}={\bar{g}}_{KA}\;m\;h\;\left(V-E_{K}\right)$$

The activation gating variable was described by:

$$m_{\infty }=\frac{1}{1+\exp\left(\frac{V+24}{-11}\right)}$$

$$\tau_{m}=3.2\;\exp\left(-\frac{V}{225}\right)$$

The inactivation gating variable was described by:

$$h_{\infty }=\frac{1}{1+\exp\left(\frac{V+65}{9}\right)}$$

$$\tau_{h}=16.4\;\exp\left(-\frac{V}{79}\right)$$

### Sodium leak channel (NaLCN)

The model for NaLCN was obtained by fitting the corresponding electrophysiological data.^72^ The current through the channel was given by (Figure S1G):

$$i_{NaLCN}={\bar{g}}_{NaLCN}\;m\;\left(V-E_{Na}\right)$$

The activation gating variable was defined by:

$$m_{\infty }=\frac{1}{1+\exp\left(\frac{V+40}{-20}\right)}$$

$$\tau_{m}=150$$

### Slow delayed rectifier potassium channel (KSR)

The model for KSR was obtained by fitting the corresponding electrophysiological data.^12^ The current through this channel was as follows (Figure S1H):

$$i_{KSR}={\bar{g}}_{KSR}n\;\left(V-E_{K}\right)$$

The activation gating particle was governed by:

$$n_{\infty }=\frac{1}{1+\exp\left(\frac{V-7.7}{-10.6}\right)}$$

$$\tau_{n}=\frac{1}{\alpha +\beta }$$

$$\alpha \left(ms^{-1}\right)=0.158\;\exp\left(\frac{V-50}{25}\right)$$

$$\beta \left(ms^{-1}\right)=0.14\;\exp\left(\frac{V+10}{-5.78}\right)$$

### Big-conductance Ca^2+^-Activated potassium channel (BK)

The model for BK was obtained by fitting the corresponding electrophysiological data.^73^ The current through the channel described by:

$$i_{BK}={\bar{g}}_{BK}\;w\;\left(V-E_{K}\right)$$

The activation gating variable was governed by (Figure S1I):

$$w_{\infty }=\frac{1}{1+\exp\left(\frac{V-V_{1/2}}{-k}\right)}$$

$$\tau_{w}=\frac{1000}{\alpha +\beta }$$

$$\alpha \left(V\right)=\alpha_{o}\;\exp\left(\frac{V-V_{o}}{38}\right)$$

$$\beta \left(V\right)=\beta_{o}\;\exp\left(\frac{V-V_{o}}{-50}\right)$$

The ${\left[Ca\right]}_{i}$ dependencies of gating kinetics were accounted for:

$$V_{1/2}=-44.19-19.55\;\ln\left({\left[Ca\right]}_{i}\right)$$

$$k=22.07+1.06\;\ln\left({\left[Ca\right]}_{i}\right)$$

$$\alpha_{o}=83.41+7.89\;\ln\left({\left[Ca\right]}_{i}\right)$$

$$\beta_{o}=843.76+78.03\;\ln\left({\left[Ca\right]}_{i}\right)$$

$$V_{o}=-89.51-21.03\;\ln\left({\left[Ca\right]}_{i}\right)$$

### Hyperpolarization-activated cyclic-nucleotide gated channel (HCN)

The model for the HCN channel was obtained by fitting the corresponding electrophysiological data.^74^ The current through the channel was defined as follows (Figure S1J):

$$i_{h}={\bar{g}}_{HCN}\;w\;\left(V-E_{rev}\right)$$

The activation gating variable of the channel was described by:

$$w_{\infty }=\frac{1}{1+\exp\left(\frac{V+89}{6.8}\right)}$$

$$\tau_{w}=\frac{1}{\alpha +\beta }$$

$$\alpha =0.0011\;\exp\left(\frac{V+71}{-19.5}\right)$$

$$\beta =0.0012\;\exp\left(\frac{V+69}{16}\right)$$

### Small-conductance Ca^2+^-Activated potassium channel (SK)

The model for SK channel was adapted from the calcium-dependent potassium channel kinetics given in Huang et al.*,*^70^ with the current through the channel given by:

$$i_{SK}={\bar{g}}_{SK}\;w\;\left(V-E_{K}\right)$$

The activation gating variable was described by (Figure S1K):

$$w_{\infty }=\frac{a_{o}{\left[Ca\right]}_{i}^{4}}{a_{o}{\left[Ca\right]}_{i}^{4}+b_{o}}$$

$$\tau_{w}=\frac{1}{a_{o}{\left[Ca\right]}_{i}^{4}+b_{o}}$$

$$a_{o}=5\times 10^{9}\;ms^{-1}mM^{-4}$$

$$b_{o}=0.01\;ms^{-1}$$

where ${\left[Ca\right]}_{i}$ was specified in mM.

### L-type calcium channel (CaL)

The model for CaL was adapted from the *L*-type calcium channel kinetics provided by Diekman et al*.*^29^ The current through this channel followed GHK conventions. The default extracellular and cytosolic calcium concentrations were set at 2 mM and 100 nM respectively.

$$i_{CaL}={\bar{g}}_{CaL}\;r\;f\;GHK\left(V,{\left[Ca\right]}_{i},{\left[Ca\right]}_{o}\right)$$

The activation gating variable was governed by (Figure S1L):

$$r_{\infty }=\frac{1}{1+\exp\left(\frac{V+36}{-5.1}\right)}$$

$$\tau_{r}=3.1$$

The inactivation gating variable, dependent on calcium and voltage, was defined as:

$$f_{\infty }=\frac{K_{1}}{K_{2}+{\left[Ca\right]}_{i}}$$

$$K1=3.93\times 10^{-5}\;mM$$

$$K2=6.55\times 10^{-4}\;mM$$

$$\tau_{f}=\exp\left(\frac{V-444}{-220}\right)$$

#### Measurements

##### Resting membrane potential and intrinsic firing frequency

The reversal potential for the leak conductance was set at −65 mV. The active currents were allowed to interact for 2 s to allow the membrane potential to settle down into a steady state, which may be spontaneously firing or silent. All measurements were obtained after this initial period of 2 s when the neuron’s RMP ($V_{RMP}$) reached a steady-state value. Since most day-like neurons manifest spontaneous action potential firing, $V_{RMP}$ was measured by median filtering the voltage traces over a period of 5 s and computing the mean of the filtered trace (Figure S2A). The intrinsic frequency ($f_{\mathrm{int}}$) was computed as the total number of spikes over a period of 5 s divided by 5.

##### Input resistance

The input resistance ($R_{in}$) was computed from the steady state voltage response to 5 hyperpolarizing current pulses between −30 pA and −70 pA, in steps of 10 pA, injected for a duration of 1 s. The steady-state voltages were plotted against the corresponding amplitude of injected current, and the slope of the linear fit to this model was taken as $R_{in}$ (Figure S2B).

##### Sag and rebound depolarization

A hyperpolarizing pulse of −30 pA was injected for a duration of 1 s (Figures S2C and S2D) to measure the sag ($V_{sag}$) and area under rebound depolarization ($A_{rebound}$). $V_{sag}$ was measured as the difference between the peak deflection ($V_{peak}$) attained following injection of the pulse and the steady state value of deflection ($V_{ss}$). $A_{rebound}$ was measured as the area under the voltage-curve, with reference to $V_{RMP}$, for a period of 150 ms following termination of hyperpolarization.

##### Spike properties

Spike properties were measured from the first action potential elicited after an initial period of 2 s. The spike amplitude ($V_{AP}$) was computed as the difference between the peak voltage attained by the spike and $V_{RMP}$ (Figure S2E). The spike width ($T_{APHW}$) was measured as the difference between the two time points at which the difference between the voltage and resting membrane potential was half of the spike amplitude (Figure S2E). The spike threshold ($V_{th}$) was measured as the voltage at the point at which the rate of change of voltage exceeded 20 V/s (Figure S2F). The amplitude of the spike after-hyperpolarization ($V_{AHP}$) was computed as the difference between the minimum voltage attained after the spike and $V_{th}$ (Figure S2G).

#### Multi-parametric multi-Objective stochastic search

To arrive at a heterogeneous population of day-like SCN neurons and to probe the manifestation of ion-channel degeneracy in the neuronal phenotypes, a multiparametric, multi-objective stochastic search (MPMOSS) algorithm was employed.^60^^,^^63^^,^^78^^,^^79^^,^^80^^,^^81^^,^^82^^,^^83^ Here, a stochastic search was performed over 13 parameters (Table 1) and the models obtained were validated using the 9 supra- and subthreshold measurements (Table 2) based on the *in vitro* electrophysiological bounds.^22^^,^^67^^,^^68^ As part of the MPMOSS algorithm, each parameter was randomly picked from a uniform distribution (Table 1). From this model, the 9 intrinsic measurements were computed and validated against the respective day-like electrophysiological bounds (Table 2). A model that satisfied all the 9 criteria for validation was used for further analysis. This process was repeated for 30,000 such unique randomized picks spanning the 13 parameters (Figure 1). Parameters were allowed to assume arbitrary values within their respective bounds, thereby avoiding artificial discretization of the parametric space.

#### Day to night transitions

To examine plasticity of individual ion channels during day to night transitions, night models were generated by picking day-like neurons from the valid model population (Figure 1). Consistent with electrophysiological observations on which channels change in what direction during the circadian oscillations,^1^^,^^11^^,^^12^^,^^13^^,^^14^^,^^15^^,^^16^^,^^22^ day-to-night transitions in these models was implemented by changing 6 parameters: the conductances of the KFR, KA, NaP, CaL, BK and NaLCN channels. While a reduction in the night conductance compared to the day conductance has been observed experimentally in case of the KFR, KA, NaP, CaL and NaLCN channels, an increase in night conductance relative to the day conductance has been observed in case of the BK channel. The signs of these experimentally observed day-to-night transition-induced changes were enforced in our model (Figure 1).

The search for transitions that would produce night-like SCN neurons from their day-like counterparts was implemented using an MPMOSS algorithm. The parametric space for the stochastic search algorithm contained sign-enforced changes in the six conductance values (Table 3) representing the transition. While the range of the distribution for ion channels that showed a reduction was restricted to (−1,0) (values higher than 1 would lead negative conductance values, and 0 represents no change), it was (0,10) in case of ion channels that would show an increase, to span a broad parametric space (Table 3). For a given model, in each iteration, a randomized percentage change was picked for each of the six conductance values within their respective bounds (Table 3). These changes were then introduced into the specific day-like model neuron, and the 9 characteristic electrophysiological properties were measured. If the neuron matches all 9 night-like properties (Table 2), the randomized transition was declared a valid transition and the model that the transition yielded was called a valid night-like model (Figure 1). This process was repeated for each of the different day-like neurons for several iterations to generate several valid night-like neurons from each day-like neuron. The parameters of valid night-like neurons were used for assessing ion-channel degeneracy, and the valid day-to-night transitions were used to investigate the manifestation of plasticity manifolds using different nonlinear dimensionality reduction techniques: *t*-distributed stochastic neighbor embedding, *t*-SNE^84^; uniform manifold approximation and projection, UMAP^85^; and Potential of Heat-diffusion for Affinity-based Trajectory Embedding, PHATE.^86^

#### Night to day transitions

An independent MPMOSS algorithm was used to arrive at valid night-to-day transitions from night-like neurons and resultant valid day-like neurons (Figure 1). The procedure and the ion-channel conductances were identical to day-to-night transitions, with the sign of transitions in individual transitions reversed for night-to-day transitions (Table 4) compared to their day-to-night counterparts. As before, the stochastic search spanned the plasticity space involving these 6 conductance values on different night-like neurons. The validation of these randomly generated models (derived from randomized transitions on different night-like neurons) was against day-like physiological properties (Table 2). Neurons that satisfied all 9 days-like properties were declared valid and the transitions that resulted in these valid day-like models were declared valid night-to-day transitions. The parametric space of valid models and associated transitions were then used to explore the manifestation of ion-channel degeneracy and plasticity manifolds.

### Quantification and statistical analysis

All simulations were performed using NEURON programming environment^66^ with an integration step size of 25 μs. All data analyses and plotting were done using custom-written scripts within IGOR Pro (Wavemetrics) and MATLAB (Mathworks) environments.

## Data Availability

• The published article includes all datasets generated or analyzed during this study. All data reported in this paper will be shared by the lead contact upon request. • A ZIP file containing the codes that were used for the simulations reported in this study is part of supplemental material. • Any additional information required to reanalyze the data reported in this paper is available from the lead contact upon request.

## Supplemental information

Document S1. Figures S1–S15

Data S1. Stimulation codes A ZIP file containing the codes that were used for the simulations reported in this study is part of supplementary material.
