# Supplementary material for: Plasticity manifolds and degeneracy govern circadian oscillations of neuronal intrinsic properties in the suprachiasmatic nucleus
Source: iScience. 2023 Mar 27;26(4):106503. doi: 10.1016/j.isci.2023.106503 (PMC10139998; doi:10.1016/j.isci.2023.106503)
Supplement: Document S1. Figures S1–S15 [file mmc1.pdf]

iScience, Volume *26*

Supplemental information

Plasticity manifolds and degeneracy govern circadian oscillations of neuronal intrinsic properties in the suprachiasmatic nucleus

Harshith Nagaraj and Rishikesh Narayanan

iScience, Volume 26

Plasticity manifolds and degeneracy govern circadian oscillations of neuronal intrinsic properties in the suprachiasmatic nucleus

Harshith Nagaraj and Rishikesh Narayanan*

*Email: rishi@iisc.ac.in

Supplementary Information

Supplementary Figure S1 2
Supplementary Figure S2 3
Supplementary Figure S3 4
Supplementary Figure S4 5
Supplementary Figure S5 6
Supplementary Figure S6 7
Supplementary Figure S7 8
Supplementary Figure S8 9
Supplementary Figure S9 10
Supplementary Figure S10 11
Supplementary Figure S11 12
Supplementary Figure S12 13
Supplementary Figure S13 14
Supplementary Figure S14 15
Supplementary Figure S15 16

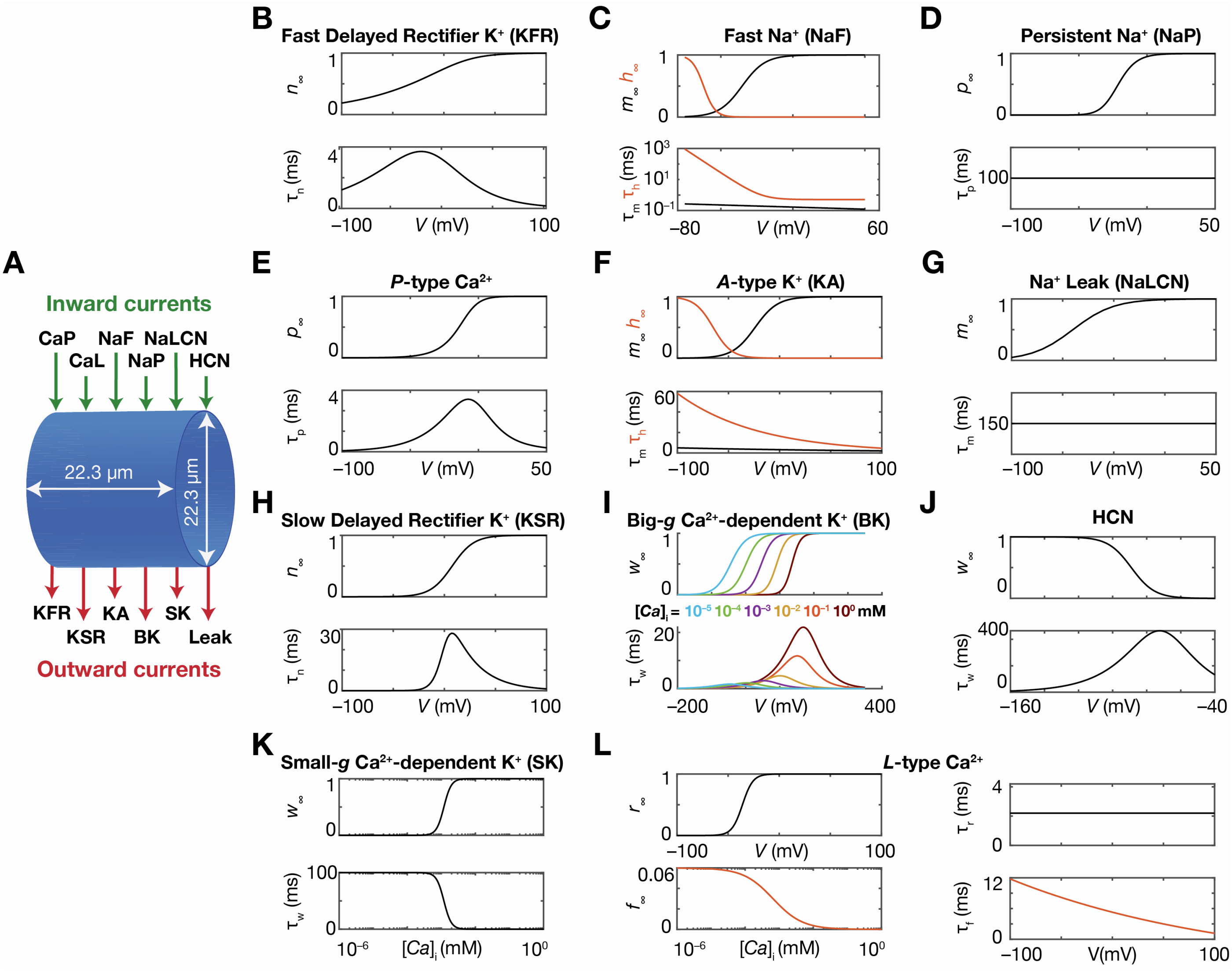

Supplementary Figure S1. Related to Figure 1.

Gating profile and kinetics of the ion channels. (*A*) Representation of the conductance-based model showing the different inward and outward channel currents. (*B–L*) Dependencies of steady-state values and the kinetics of the gating variables associated with the different ion channel conductances in the model. In panels C, F, and L, black and orange lines represent activation and inactivation gating variables, respectively.

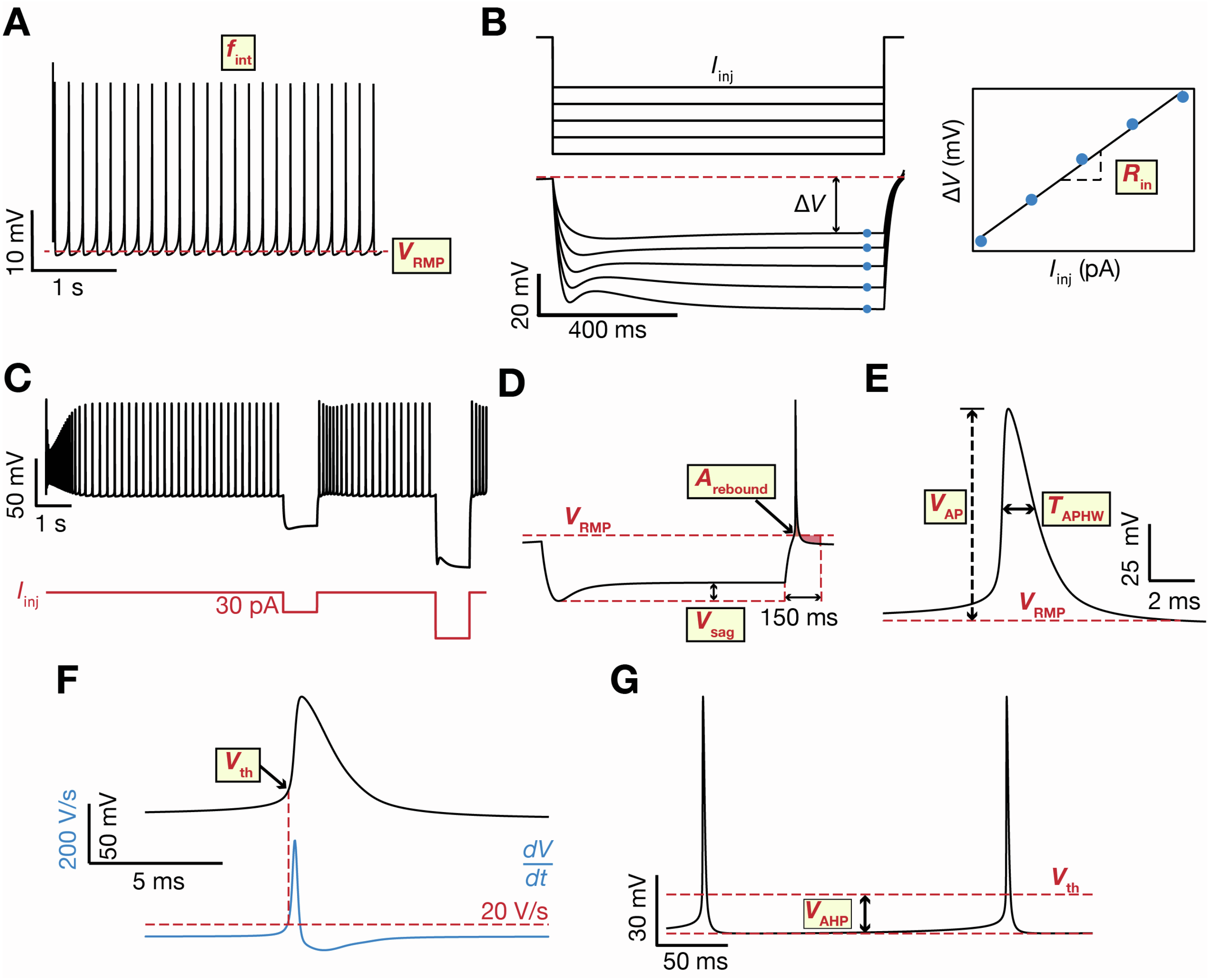

Supplementary Figure S2. Related to Figure 2.

Electrophysiological measurements used to characterize and validate the models. (*A*) The resting membrane potential (V_{RMP}, red line) was measured as the mean of the median-filtered voltage trace computed over a duration of 5 s. The intrinsic frequency (f_{int}) was computed as the total number of spikes over a period of 5 s divided by 5. (*B*) The input resistance (R_{in}) was measured as the slope of the linear fit to the steady state voltage deflection obtained by injecting 5 hyperpolarizing current pulses (–30 pA to –70 pA, in steps of 10 pA) for a period of 1 s. (*C*) Voltage response of an example neuron (black) to current injections (red). The neuron is spontaneously active, with a characteristic sag and rebound depolarization seen due to the injection of hyperpolarizing current pulses. (*D*) The sag (V_{sag}) and rebound depolarization (V_{rebound}) were measured after injecting a hyperpolarizing current pulse of –30 pA for a duration of 1 s. The sag was the difference between the steady state voltage and the minimum voltage obtained after injecting the current pulse. Rebound depolarization was the area under the voltage-time curve (shaded in red) over a period of 150 ms after the current injection. (*E*) The spike amplitude (V_{AP}) was quantified as the difference between the maximum voltage attained by the spike with reference to V_{RMP}. Spike width (T_{APHW}) was quantified as the difference between the two timepoints when the voltage attained was half the spike amplitude with reference to V_{RMP}. (*F*) The spike threshold (V_{th}) was measured as the voltage attained at the point when the rate of change of voltage with time (dV/dt, blue) exceeded 20 V/s. (*G*) The spike after-hyperpolarization (V_{AHP}) was quantified as the difference between the minimum voltage attained within 40 ms after the spike attained its maximum value (lower red line) and V_{th}.

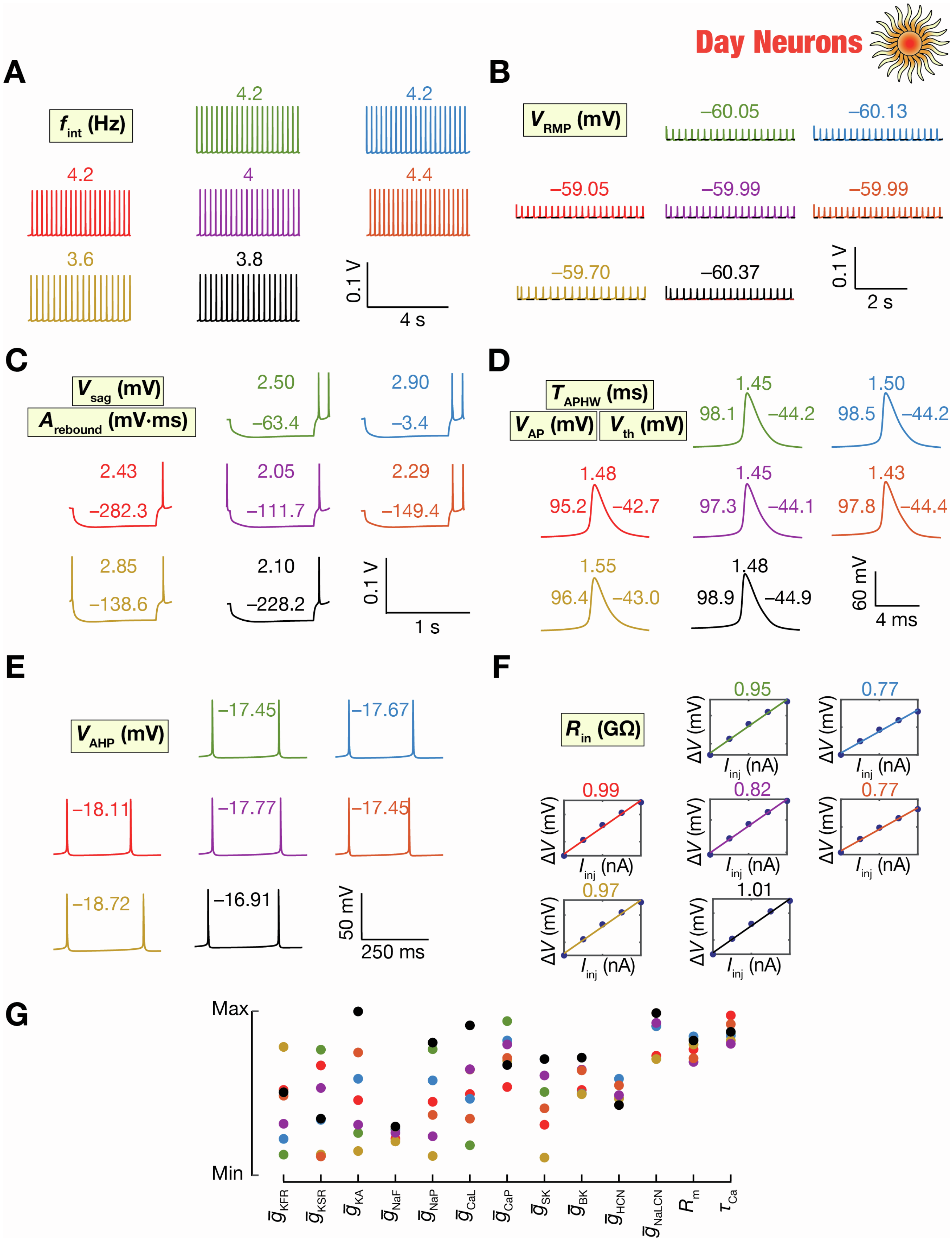

Supplementary Figure S3. Related to Figure 2.

Parametric degeneracy among day-like SCN neurons showing similar characteristic physiological properties. (*A*–*F*) Measurements of 7 different day-like SCN neurons (picked from the 128 shown in Fig. 2) which exhibit similar characteristic physiology. Different colors represent different neurons, and the values of the corresponding measurements are shown above the traces and were similar across the 7 neurons. (*G*) The 7 models showed pronounced heterogeneity in their parametric values, with no clustering in parameters, providing an illustration of the expression of degeneracy.

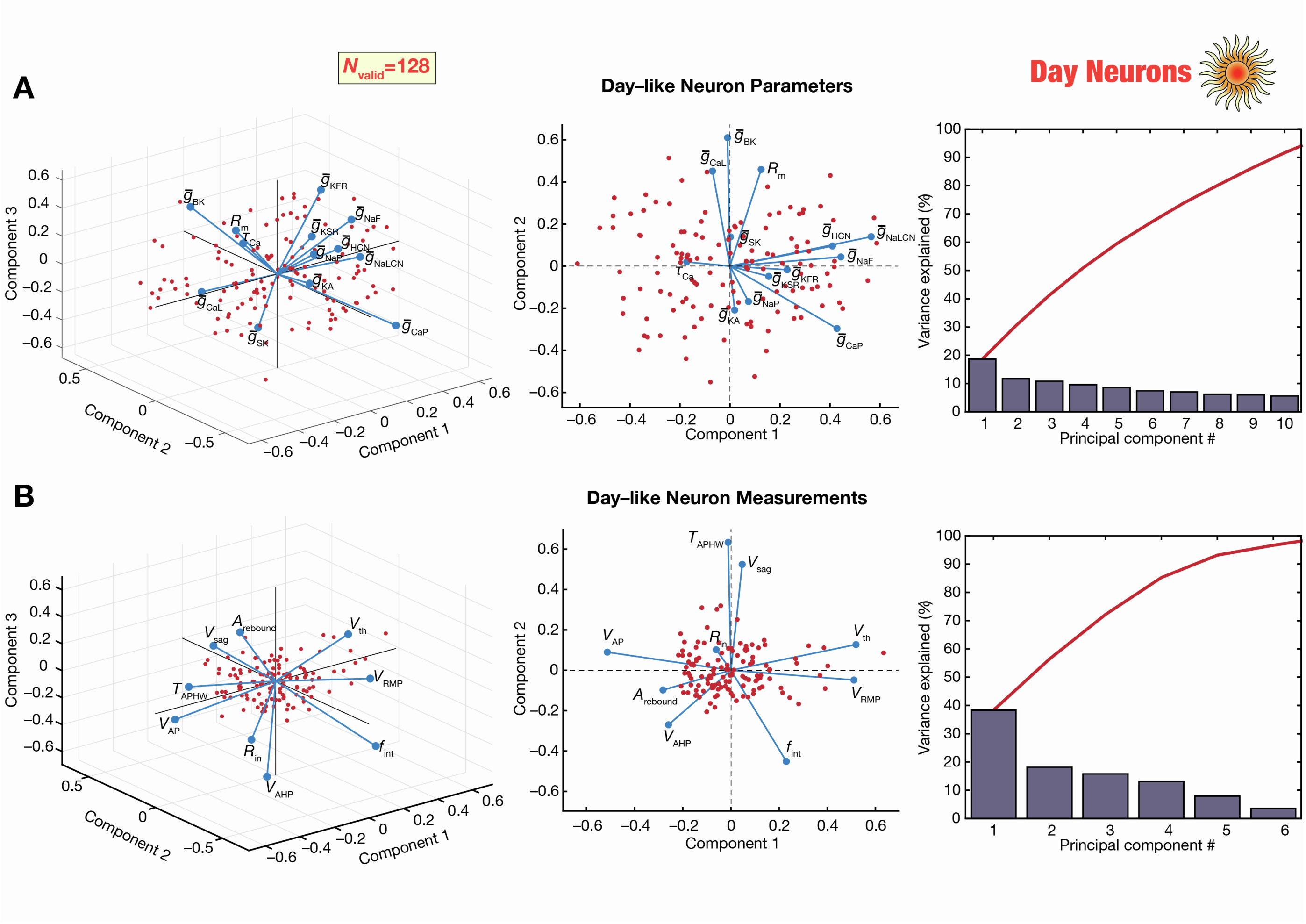

Supplementary Figure S4. **Related to Figure 3.**

Principal component analyses of parameters and measurements of the heterogeneous day-like neuronal population. (*A*) *Left–Middle*, Representation of the parameters of the 128 day-like neurons on a reduced 3D (left) or 2D (middle) space obtained from PCA. The axes represent the first 3 (left) or 2 (middle) principal components, with each point (in red) representing a distinct day-like neuron. Blue lines represent the loadings of the different parameters. *Right*, Scree plot representing the population variance explained by successive principal components. Note that the cumulative explanatory power of the first three principal components was <40% of variance explained. (*B*) Same as panel *A*, but for measurements of the 128 day-like neurons. Note that the cumulative explanatory power of the first three principal components was <70% of variance explained.

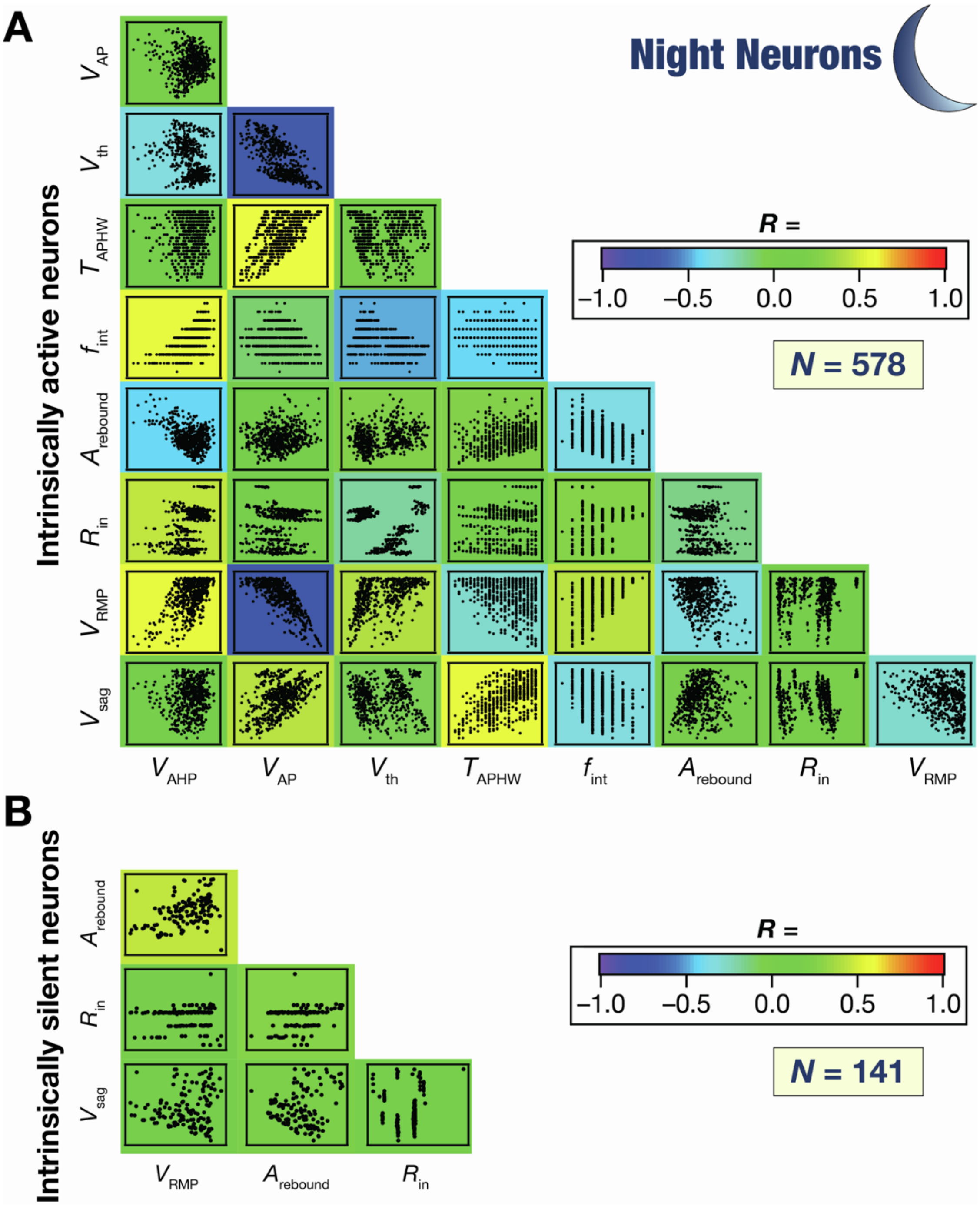

Supplementary Figure S5. Related to Figure 4.

A majority of pairwise correlations between measurements from the night-like neuronal model population were weak. (*A*–*B*) Pair-wise correlations between the different measurements used to characterize and validate night-like neuronal models. The scatter plots are overlaid on top of the color-coded scatter plot matrix showing the respective Pearson's correlation coefficient values. Panels *A* and *B* show measurement correlations from intrinsically active (*A*) and intrinsically silent (*B*) night neurons. The matrix in *B* does not include action potential measurements because these neurons are intrinsically silent, and action potential properties were derived from spontaneous action potentials.

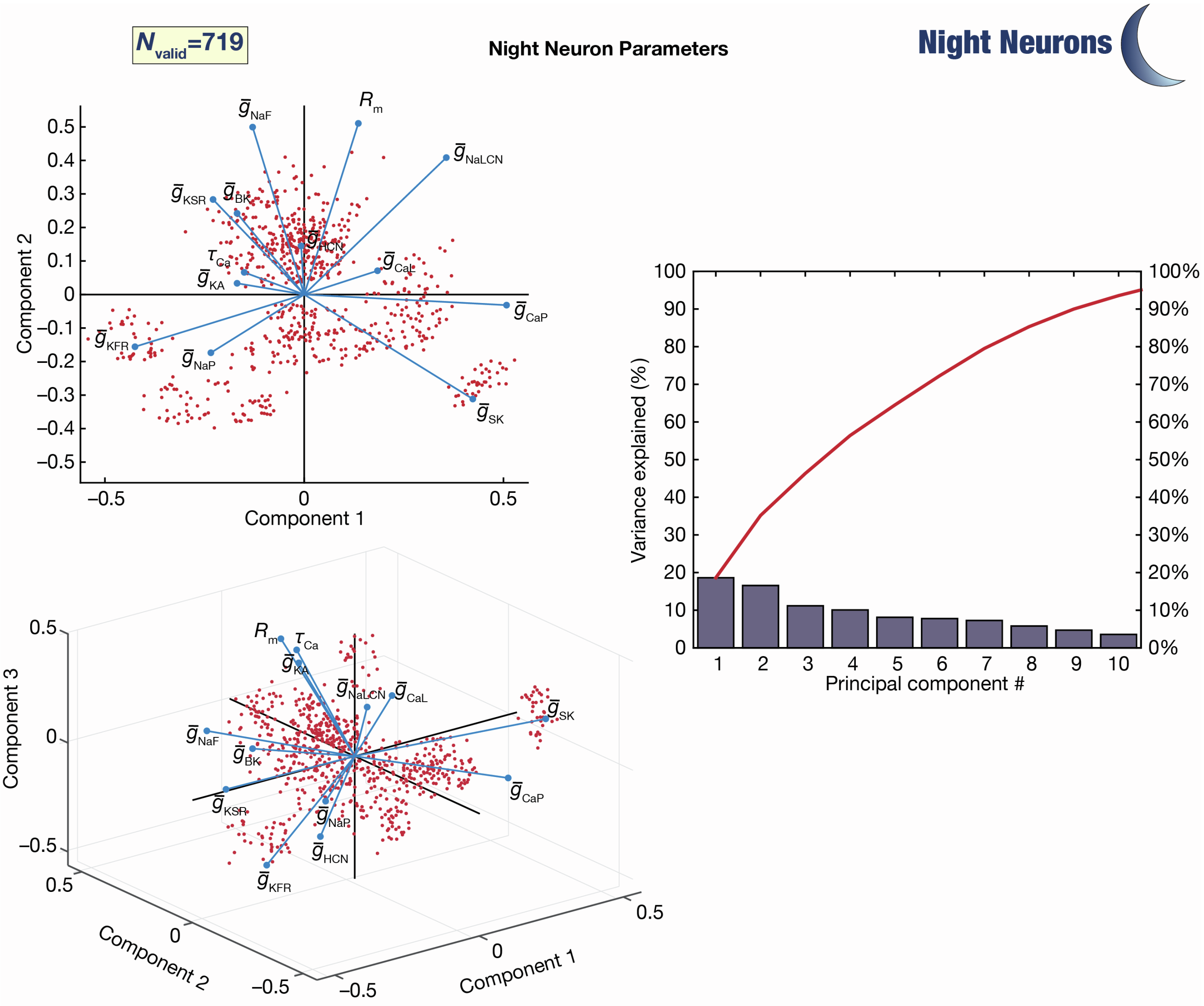

Supplementary Figure S6. **Related to Figure 5.**

Principal component analyses of parameters of the heterogeneous night-like neuronal population. *Left*, Representation of the parameters of the 719 night-like neurons on a reduced 2D (top) or 3D (bottom) space obtained from PCA. The axes represent the first 2 (top) or 3 (bottom) principal components, with each point (in red) representing a distinct night-like neuron. Blue lines represent the loadings of the different parameters. *Right*, Scree plot representing the population variance explained by successive principal components. We noted that the clustering observed in the parametric space reflected the origin day-like neuron (in Fig. 4*B*–*C*) from which these night-like neurons transitioned from. Note that the cumulative explanatory power of the first three principal components was ~40% of variance explained.

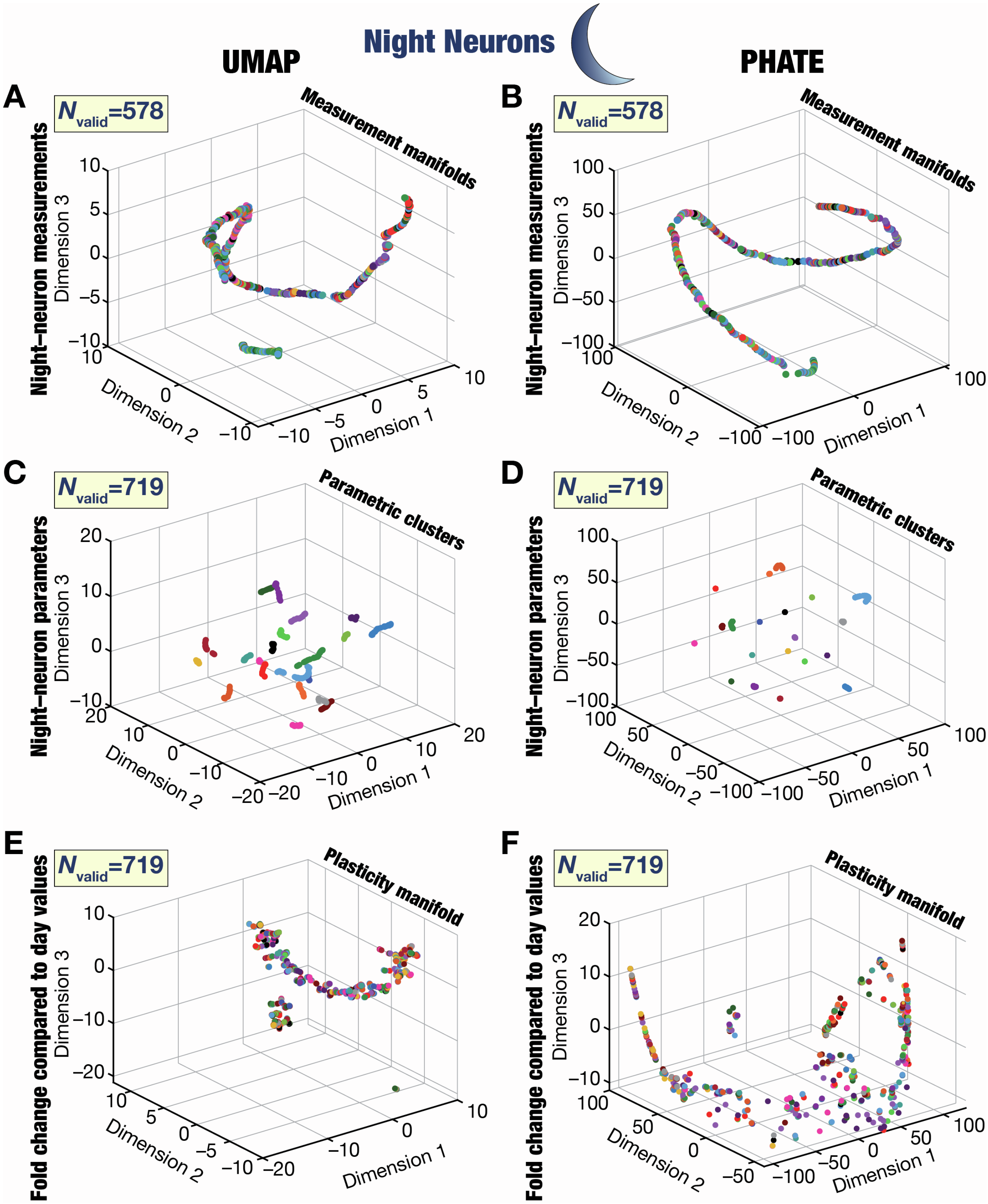

Supplementary Figure S7. Related to Figure 6.

Conclusions about reduced dimensionality spaces of night-like neurons were invariant to the specific dimensionality reduction technique used. Visualization of the measurement (*A*–*B*), parametric (*C*–*D*), and plasticity (*E*–*F*) spaces associated with the 719 night-like neuron measurements using dimensionality reduction analyses performed with UMAP (*A*, *C*, *E*) or PHATE (*B*, *D*, *F*). Each point represents a distinct night-like neuron and are color-coded (20 colors) with reference to the day-like neuron from which it transitioned. In Panels *A*–*B*, analyses were performed only the 578 intrinsically active night-like neurons because the silent neurons didn't elicit action potentials making action potential measurements infeasible. The other panels are for all 719 valid night-like neurons.

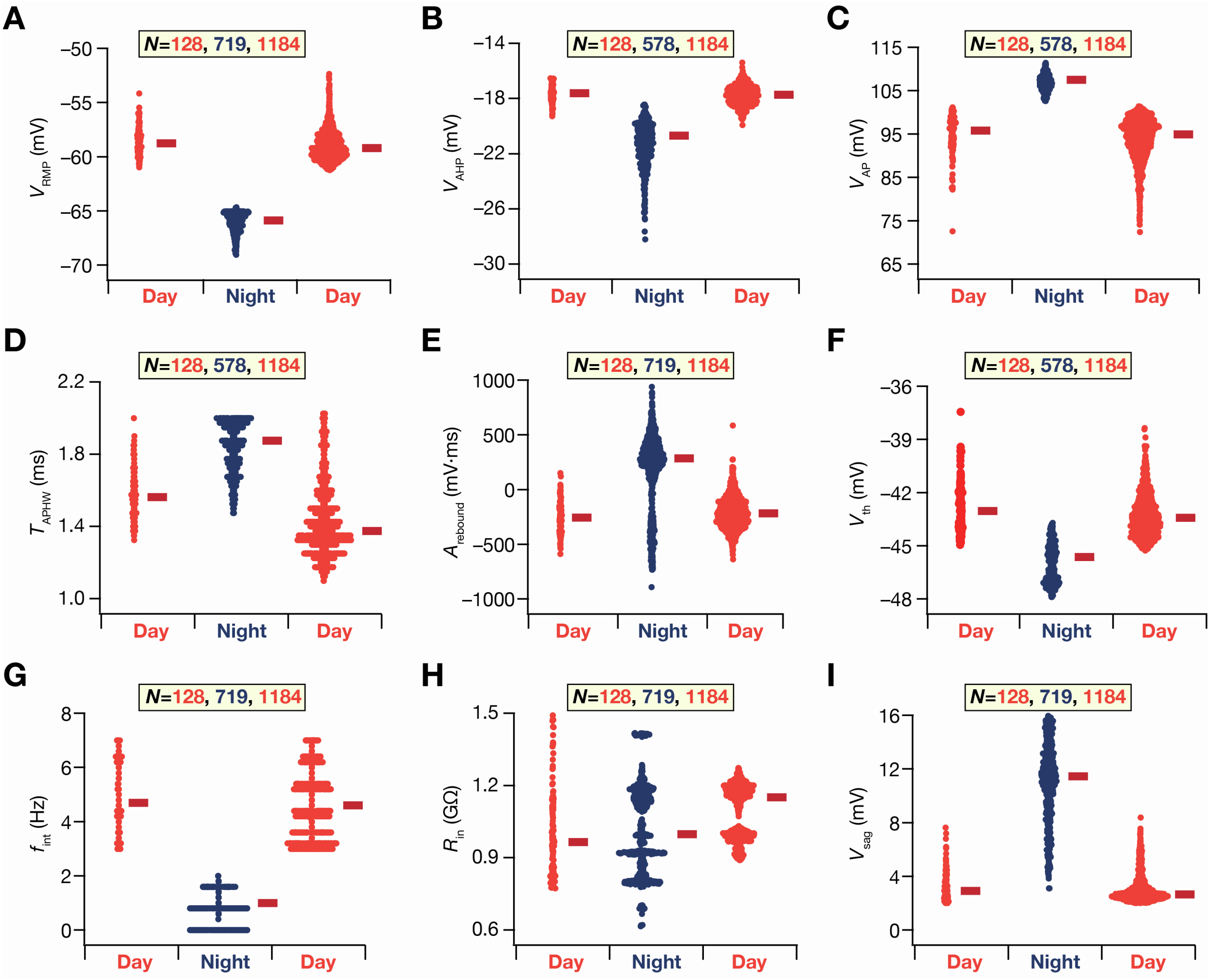

Supplementary Figure S8. **Related to Figure 7.**

Measurement evolutions across a full circadian cycle showing the original population of day-like neurons, the transitioned populations of night-like neurons, and the day-like neurons after a full cycle. Bee-swarm plots of all 9 physiological measurements of the different day (red) and night (blue) neurons. Figure 1 provides the flowchart of how day neurons and day-to-transition as well as night-to-day were executed within our modeling. The red line represents the median value. A clear difference in measurements from day and night neurons may be observed (*cf.* Table 2).

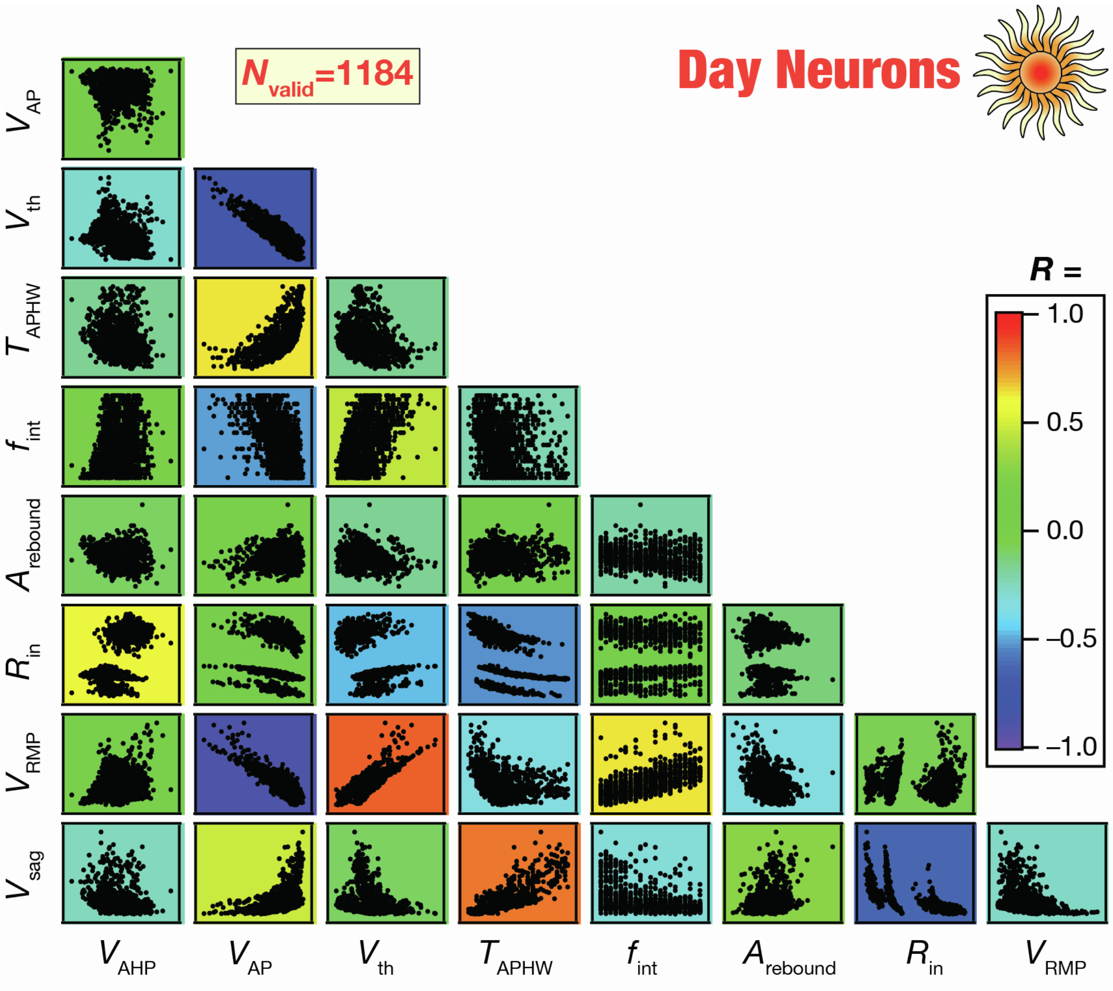

Supplementary Figure S9. Related to Figure 7.

Pairwise correlations between measurements from the day-like neuronal model population derived by physiological transitions from night-like neurons. Pairwise correlations between the different measurements used to characterize and validate day-like neuronal models. The scatter plots are overlaid on top of the color-coded scatter plot matrix showing the respective Pearson's correlation coefficient values.

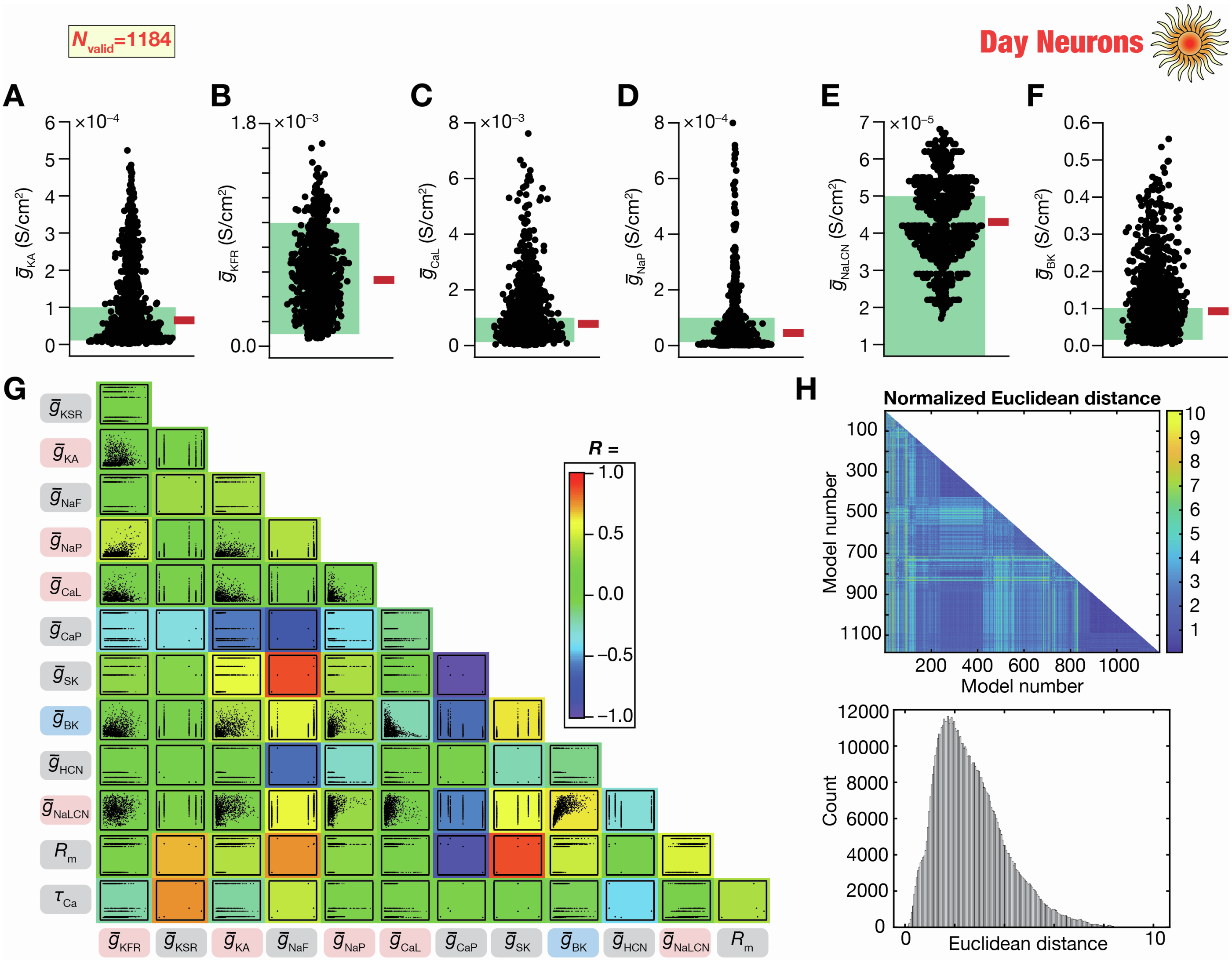

Supplementary Figure S10. Related to Figure 7.

Heterogeneous distribution and weak pairwise correlations of parameters from day-like SCN neurons derived from night-like neurons. (*A*–*F*) Bee-swarm plots of the parameters from 1184 different SCN day-like neurons shown in Fig. 7. Red bars represent the median values. Green shaded portions represent the set range of the respective parameters in the original day-neuron population (Table 1). (*G*) Scatter-plot matrix between the different parametric values of the 1184 day-like SCN models shown in Fig. 7. The background color represents the value of the Pearson correlation coefficient between the different parameters. Ion channels represented with blue and red background show reductions and increases during night-to-day transitions, respectively. Parameters with gray background did not change during day-to-night or night-to-day transitions, and thus do not change from their respective day neurons (*N*=5). (*H*) Heterogeneities in model parameters quantified with normalized Euclidean distances. The matrix represents the pairwise distance between the parametric vectors defining the 1184 models. The bottom plot shows the histogram of the values in the distance matrix. The singularity of the associated covariance matrix precluded computation of the Mahalanobis distances for the day-like neurons.

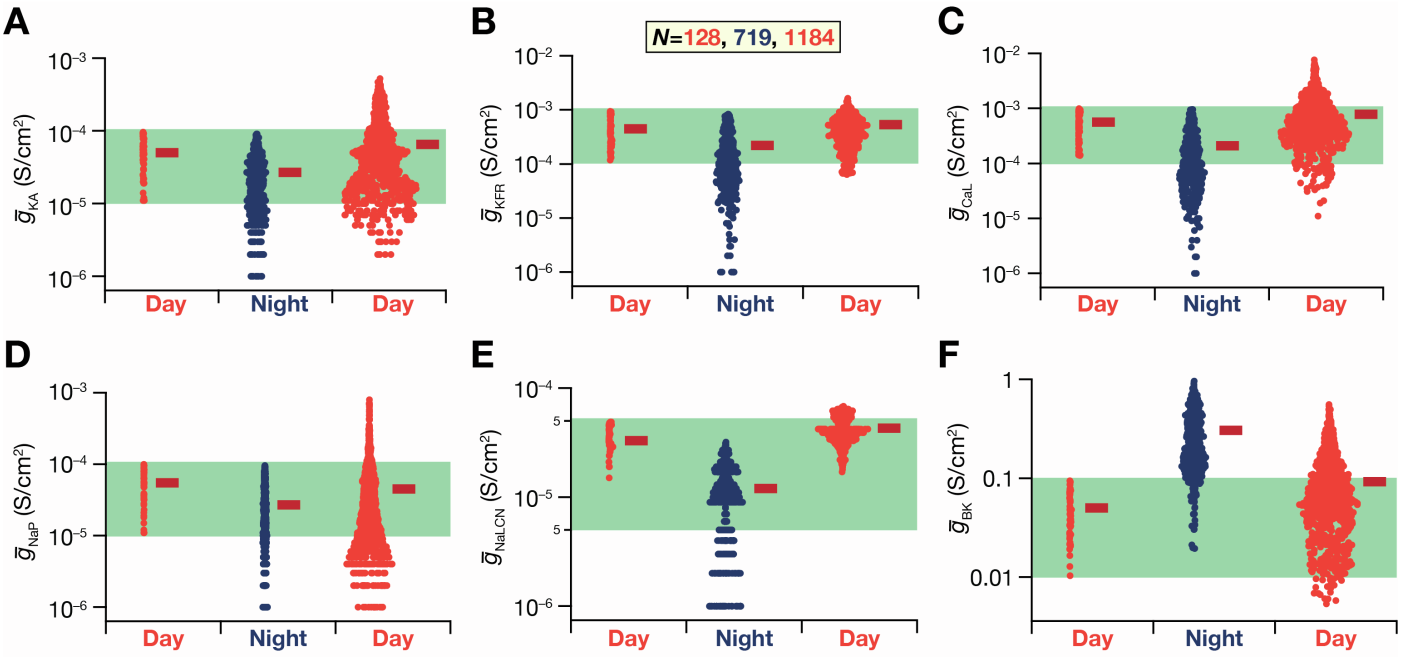

Supplementary Figure S11. Related to Figure 7.

Evolutions of the plastic ion-channel conductances across a full circadian cycle showing the original population of day-like neurons, the transitioned populations of night-like neurons, and the day-like neurons after a full cycle. Bee-swarm plots of the plastic parameters that were subjected to sign-enforced change during the circadian oscillations of the day (red) and night (blue) neurons. Red line represents the median. The shaded region (green) represents the bounds of the MPMOSS used to generate the initial day population (from Table 1). Figure 1 provides the flowchart of how day neurons, day-to-night as well as night-to-day transitions were executed within our modeling framework.

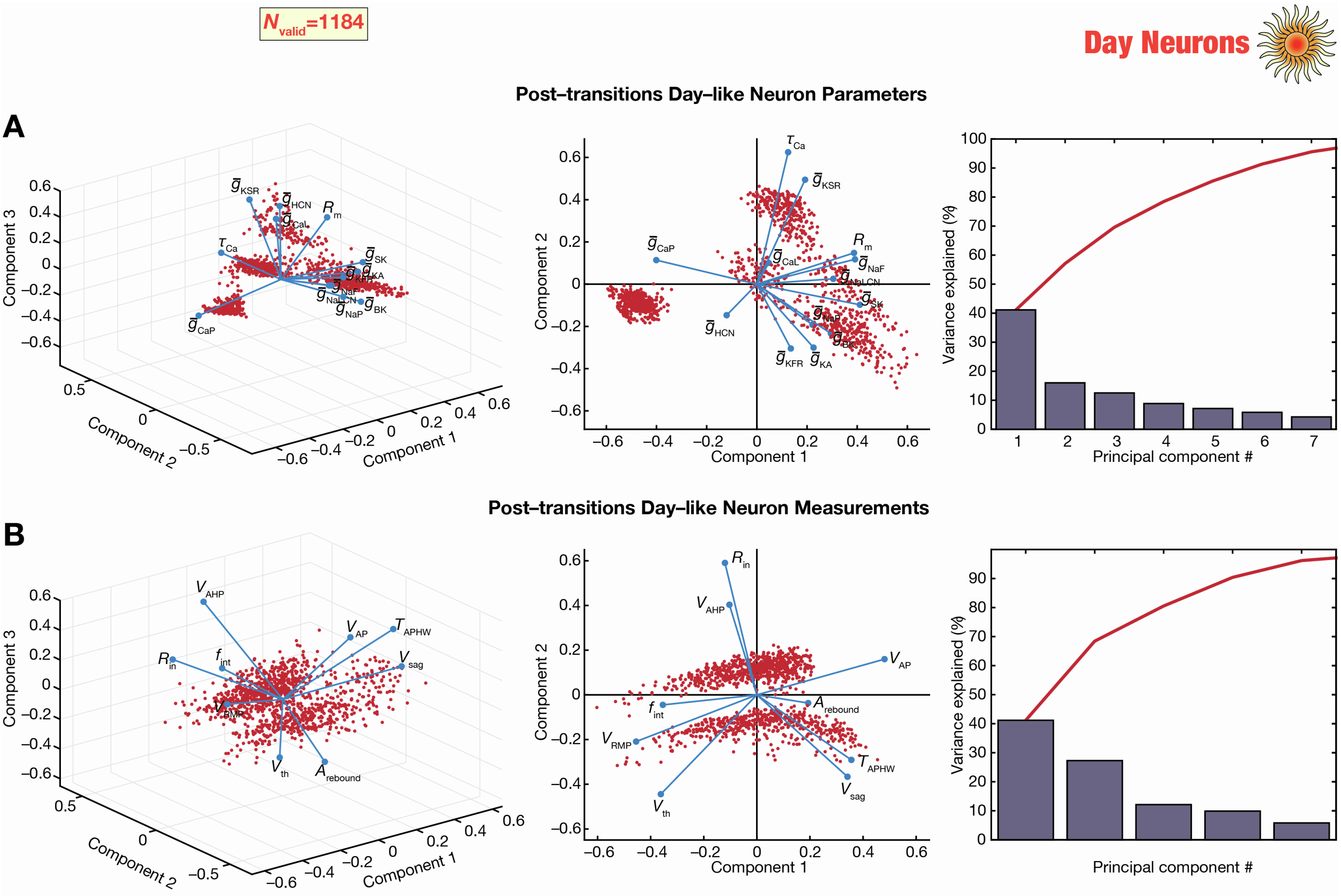

Supplementary Figure S12. **Related to Figure 7.**

Principal component analyses of parameters and measurements of the heterogeneous day-like neuronal population after one full circadian cycle. (*A*) *Left–Middle*, Representation of the parameters of the 1184 day-like neurons on a reduced 3D (left) or 2D (middle) space obtained from PCA. The axes represent the first 3 (left) or 2 (middle) principal components, with each point (in red) representing a distinct day-like neuron. Blue lines represent the loadings of the different parameters. We noted that the clustering observed in the parametric space reflected the origin night-like neuron (in Fig. 7*B*) from which these day-like neurons transitioned from. *Right*, Scree plot representing the population variance explained by successive principal components. Note that the cumulative explanatory power of the first three principal components was ~60% of variance explained. (*B*) Same as panel *A*, but for measurements of the 1184 day-like neurons. We noted that the clustering observed in the measurement space reflected the origin night-like neuron (in Fig. 7*A*) from which these day-like neurons transitioned from, especially in terms of the two populations of origins neurons with different input resistances (observed in Fig. 7*A*, Fig. 7C, and in the loadings for R_{in} in this panel). Note that the cumulative explanatory power of the first three principal components was ~70% of variance explained.

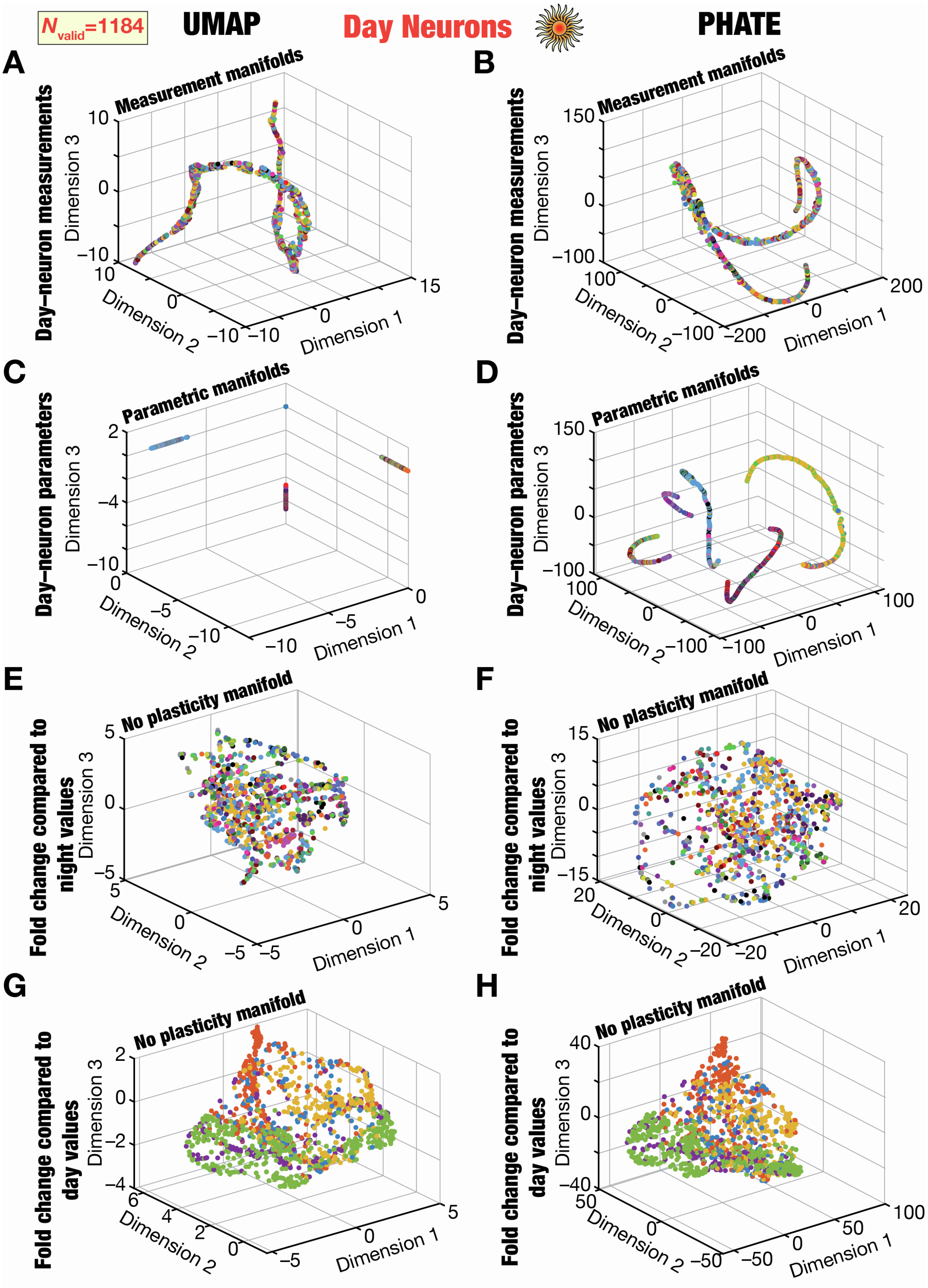

Supplementary Figure S13. Related to Figure 8.

Conclusions about reduced dimensionality spaces of night-like neurons were invariant to the specific dimensionality reduction technique used. Visualization of the measurement (*A*–*B*), parametric (*C*–*D*), and plasticity (*E*–*F*) spaces associated with the 1184 day-like neuron measurements (obtained after day-to-night and a subsequent night-to-day transitions) using dimensionality reduction analyses performed with UMAP (*A*, *C*, *E*, *G*) or PHATE (*B*, *D*, *F*, *H*). Each point represents a distinct day-like neuron and are color-coded with reference to the night-like neuron (*A*–*F*; 26 colors) or the original day-like neuron (*G*–*H*; 5 colors) from which it transitioned.

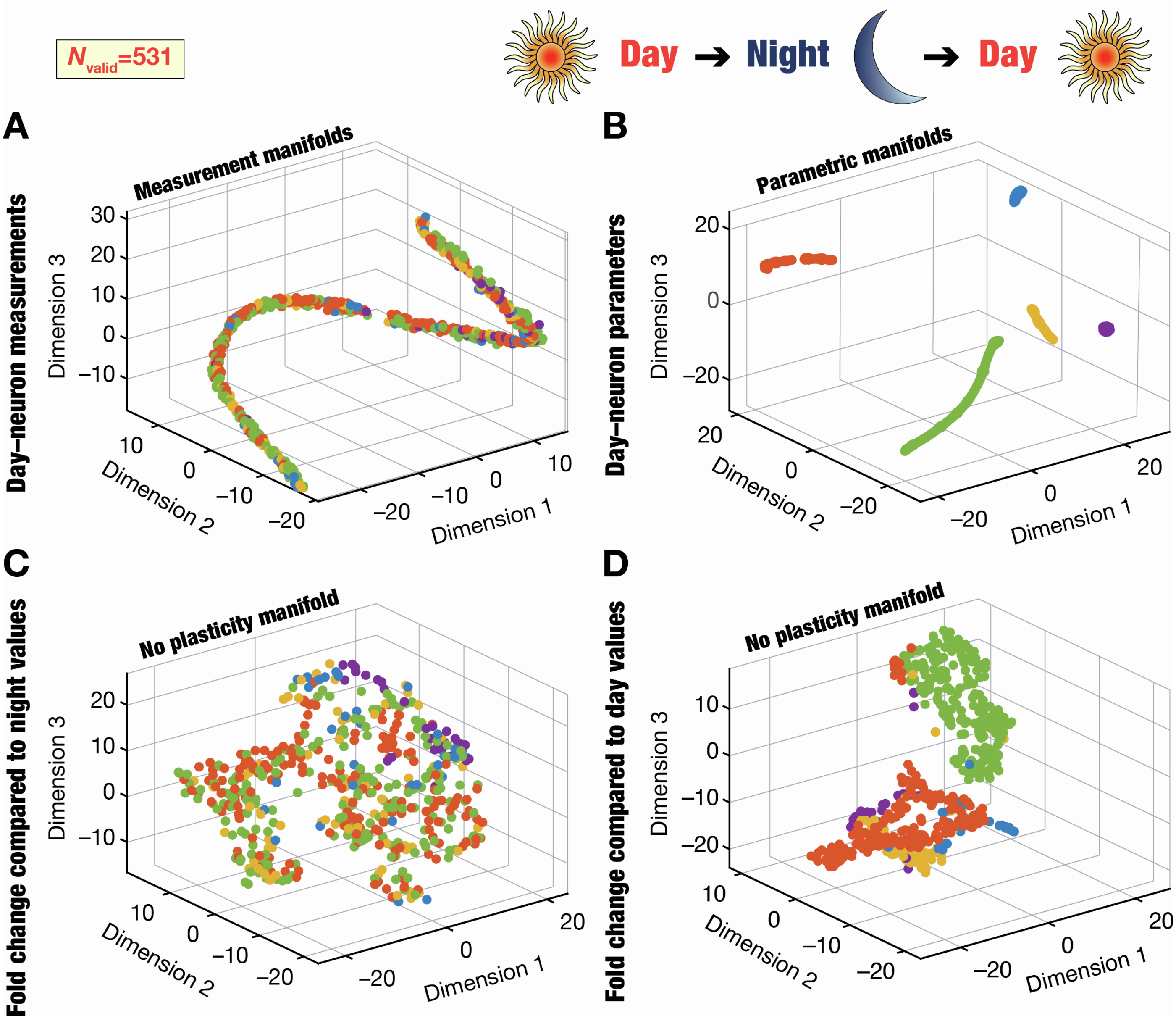

Supplementary Figure S14. Related to Figure 8.

Conclusions about reduced dimensionality spaces of night-like neurons held when transitions were restricted to distinct day-like and subsequent night-like neuronal ancestors. Visualization of the measurement (*A*), parametric (*B*), and plasticity (*C*) spaces associated with 531 day-like neuron measurements (obtained after day-to-night and a subsequent night-to-day transitions) using dimensionality reduction analyses performed with *t*-SNE. These day-like neurons were obtained from 5 night-like neurons, each of which were derived from a distinct day-like neuron. The day-to-night and night-to-day transitions were implemented using the same algorithm shown in Figure 1. Each point represents a distinct day-like neuron and are color-coded with reference to the night-like neuron (*A*–*F*; 5 colors) or the original day-like neuron (*G*–*H*; 5 colors) from which it transitioned from.

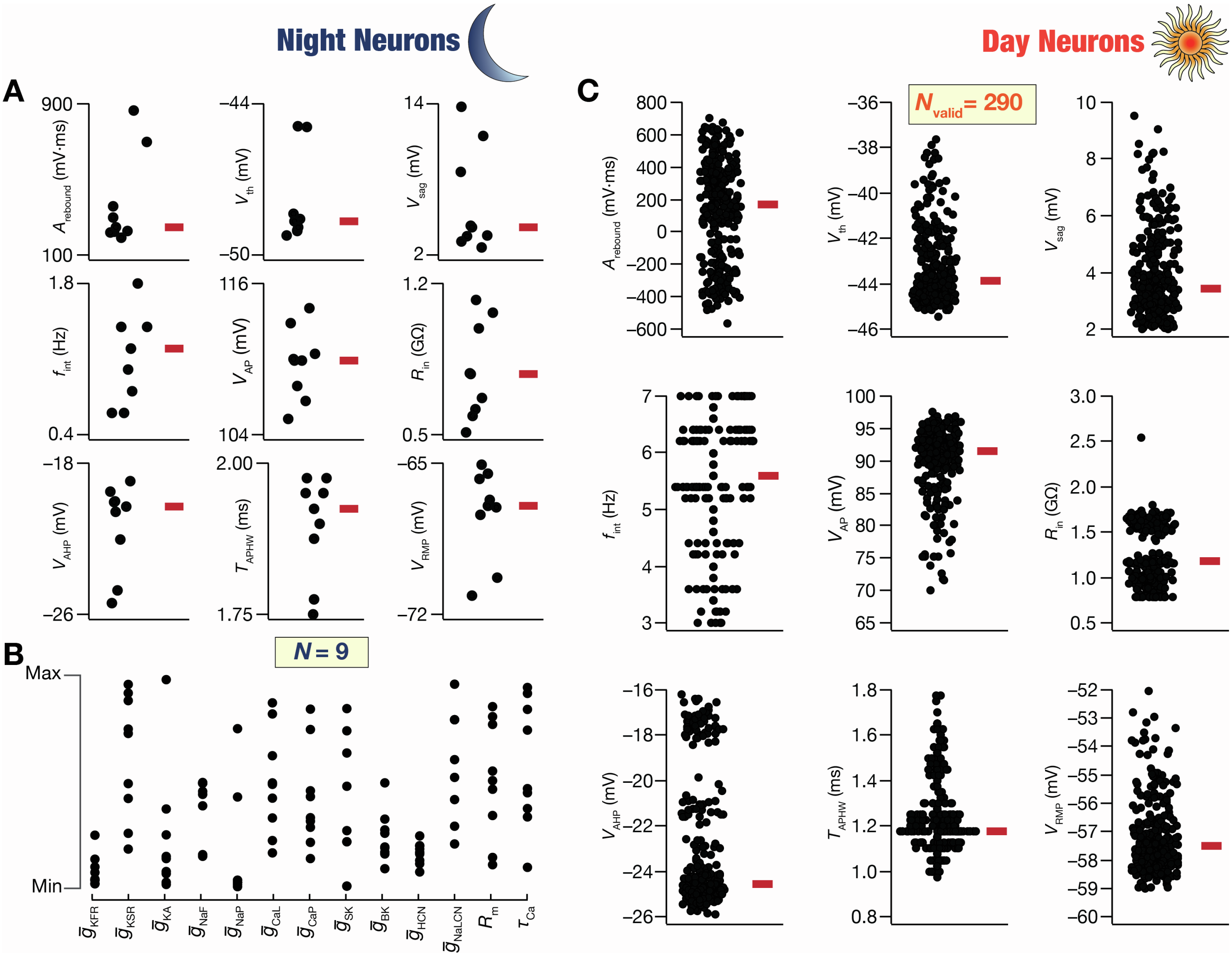

Supplementary Figure S15. Related to Figure 9.

Heterogeneous distribution of measurements from day-like SCN neurons derived through unbiased search of transitions from the *de novo* population of night-like neurons. (*A*–*B*) Bee-swarm plots of measurements (*A*) and parameters (*B*) of the 9 night-like SCN neuron models that were subjected to the night-to-day transition (see Fig. 9*A*). The transition was implemented through a modified MPMOSS algorithm that was employed to perform an unbiased search on the plasticity space. The plasticity space accounted for the physiological direction of changes in the six ion channels (shown in Fig. 9*A*) that are known to undergo plasticity during circadian oscillations. The widespread distribution of the measurements and the parameters of the 9 night-like neurons may be noted. (*C*) Bee-swarm plots of the measurements from 290 different SCN day-like neurons derived from the 9 night-like neurons. Red bars represent the median values. The validation process for obtaining day-like neurons employed established electrophysiological bounds on each measurement (Table 2).
